# Local Climate Heterogeneity Shapes Population Genetic Structure of Two Undifferentiated Insular *Scutellaria* Species

**DOI:** 10.3389/fpls.2017.00159

**Published:** 2017-02-10

**Authors:** Huan-Yi Hsiung, Bing-Hong Huang, Jui-Tse Chang, Yao-Moan Huang, Chih-Wei Huang, Pei-Chun Liao

**Affiliations:** ^1^Department of Life Science, National Taiwan Normal UniversityTaipei, Taiwan; ^2^Department of Entomology, National Taiwan UniversityTaipei, Taiwan; ^3^Division of Silviculture, Taiwan Forestry Research InstituteTaipei, Taiwan

**Keywords:** climate heterogeneity, genetic variation, precipitation, *Scutellaria*, taxonomy, typhoon

## Abstract

Spatial climate heterogeneity may not only affect adaptive gene frequencies but could also indirectly shape the genetic structure of neutral loci by impacting demographic dynamics. In this study, the effect of local climate on population genetic variation was tested in two phylogenetically close *Scutellaria* species in Taiwan. *Scutellaria taipeiensis*, which was originally assumed to be an endemic species of Taiwan Island, is shown to be part of the widespread species *S. barbata* based on the overlapping ranges of genetic variation and climatic niches as well as their morphological similarity. Rejection of the scenario of “early divergence with secondary contact” and the support for multiple origins of populations of *S. taipeiensis* from *S. barbata* provide strong evolutionary evidence for a taxonomic revision of the species combination. Further tests of a climatic effect on genetic variation were conducted. Regression analyses show nonlinear correlations among any pair of geographic, climatic, and genetic distances. However, significantly, the bioclimatic variables that represent the precipitation from late summer to early autumn explain roughly 13% of the genetic variation of our sampled populations. These results indicate that spatial differences of precipitation in the typhoon season may influence the regeneration rate and colonization rate of local populations. The periodic typhoon episodes explain the significant but nonlinear influence of climatic variables on population genetic differentiation. Although, the climatic difference does not lead to species divergence, the local climate variability indeed impacts the spatial genetic distribution at the population level.

## Introduction

Species delimitation is a subjective judgment of the level of divergence between organisms despite using objective evidences, while exploring the mechanisms causing genetic divergence is a more objective approach to describing the process of species divergence. In the genic species concept (Wu, 2001), introgression should be heterogeneous across the genome during the speciation process. As time goes by, the segregation of genes will become more and more obvious, and whenever the balancing selection that maintains the shared adaptive polymorphisms in both species is relaxed, random genetic drift may cause decreasing numbers of highly introgressed genes (cf. de Lafontaine et al., 2015). Under this process, an allopatric distribution is not a necessary criterion for speciation, and the proportion of the permeable loci could therefore be an indicator for evaluating the completeness of speciation (Wu, 2001; de Lafontaine et al., 2015).

Interspecific gene flow between two genetically distinct species could be the result of two contrasting evolutionary hypotheses. The first hypothesis is ecologically based speciation with continuous gene flow during the speciation process. When species occur in sympatry, ecological selection should act to maintain their divergence. An alternative hypothesis addresses the introgression following secondary contact after the completion of speciation. Such ecologically divergent species might be able to interbreed with each other during the onset of divergence and secondary contact (Pinho and Hey, 2010), and recent secondary contact might also reduce the number of adaptive divergent loci and result in high genetic convergence (Strasburg et al., 2012). When the introgression occurs after secondary contact, neutral loci can be more easily co-vary to reflect the history of differentiation than the selected loci (i.e., the genetic—environment association) (Bierne et al., 2013). Under both hypothesis, niche of two recently divergent species might be either bear some similarity from ancestor to descendant through time due to endogenous factor constraint (Losos, 2008), or be constrained through the mechanism of phylogenetic niche conservatism (Ackerly, 2003; Wiens, 2004; Pyron et al., 2015). Consequently, the ancestral fundamental niche characteristics will be retained in species with different ecological environments.

In addition to its effect on species divergence, environmental heterogeneity could also accelerate genetic change and divergence among populations if they lack sufficient compensation for gene flow (cf. Fountain et al., 2016). Temperature and water availability could be the main factors influencing the distribution range of plant species (Jump and Penuelas, 2005). Differential responses in genetic variation to the climate could be at a much finer geographic scale than might be expected (Owuor et al., 1997; Li et al., 1999; Huang et al., 2002). The local climate is a complicated set of different environmental elements. Temperature and precipitation are the two main dimensions of local climate change, and have also been suggested as the main niche characteristics related to the occurrence, abundance, and distribution of species (Grinnell, 1917; Gavin and Hu, 2006; Dormann, 2007). Identifying the specific climatic variables that affect the spatial and temporal genetic distribution is important for understanding how plants adapt and respond to global climate change (Carta et al., 2016; Huang C. L. et al., 2016; Vidigal et al., 2016).

In Taiwan, the short distance from continental Asia (180 km on average) and the recency of island formation (< 5 Mya; Chi et al., 1981; Teng, 1996; Wang et al., 2002) together with its ragged terrain have created a large number of recently speciated endemic taxa. For instance, six of the eight *Scutellaria* species are endemic to Taiwan Island. The high endemism in this group of species is suggested to be a consequence of both multiple origins and *in situ* diversification (Chiang et al., 2012a). *Scutellaria indica*, one of the two non-endemic species in Taiwan, is suggested to have colonized Taiwan from China through the north, and subsequently to have split off *S. tashiroi, S. austrotaiwanensis*, and *S. playfairii*, which are genetically (Chiang et al., 2012a) and morphologically (Yamazaki, 1992; Hsieh and Huang, 1995, 1997) similar to each other, via a sequence of episodes of dispersal and fragmentation corresponding to the interglacial–glacial cycles (Chiang et al., 2012a). Another endemic species, *S. taiwanensis*, is phylogenetically distinct from the others and has a restricted distribution in southern Taiwan (Chiang et al., 2012a). A recently discovered endemic species, *S. hsiehii*, is largely divergent from the other Taiwanese species in morphology and phenology and was only found on a logging trail in central Taiwan (Hsieh, 2013). *Scutellaria hsiehii* is another phylogenetic lineage distinct from any other Taiwanese species inferred by a neutral nuclear gene *CHACLONE SYNTHASE* (Huang B.-H. et al., 2016).

**Figure 1:**
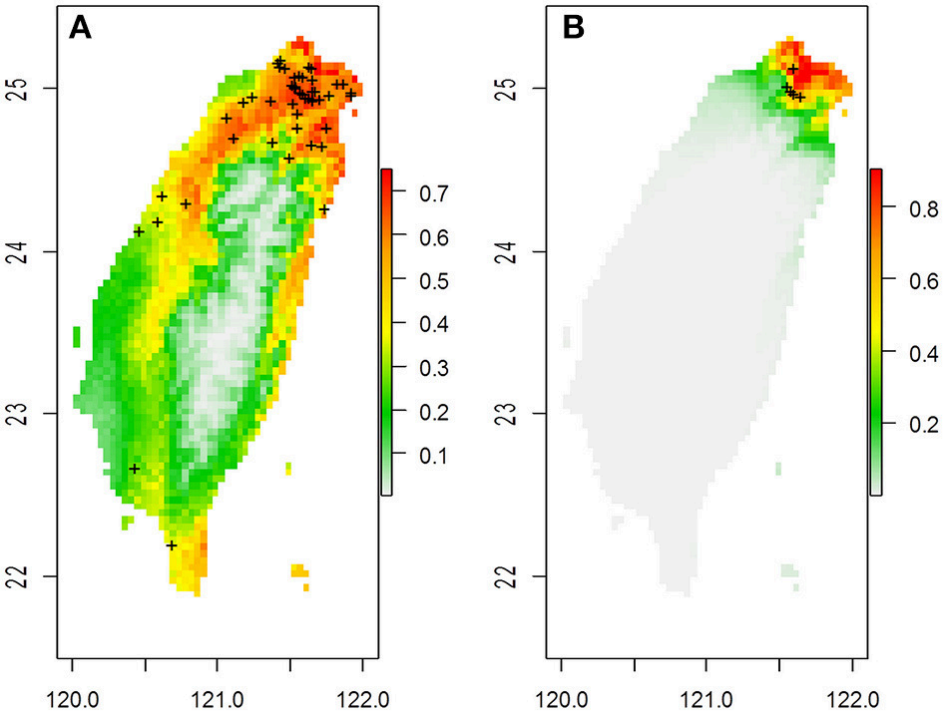
**The predicted spatial distribution of two ***Scutellaria*** species in Taiwan based on 19 bioclimatic variables in (A)** *S. barbata* and **(B)** *S. taipeiensis*. Crosses on the maps are the distribution of specimen records.

*Scutellaria barbata* and *S. taipeiensis* (hereinafter referred to as *bar* and *tpe*, respectively) are two morphologically similar species with overlapping distributional ranges (Figure 1 and Supplementary Figure 1). *Scutellaria barbata* is a widespread Asian species while *tpe* is endemic to northern Taiwan (Huang et al., 2003). They can be recognized as distinct species under the morphological species concept due to slight but constant morphological differences (Huang et al., 2003). However, such slight morphological differences with overlapping geographical distribution imply similar niches attributing to convergent selective pressures. The chloroplast and nuclear DNA sequences reveal that these two species diverged very recently but are distinct from other Taiwanese species (Chiang et al., 2012a). In contrast with the eastern and southern distribution of other Taiwanese *Scutellaria* species, *bar* and *tpe* are sparsely distributed in western and northern Taiwan. This, together with their similar genetic composition and overlapping geographic distribution, suggests the lineage of *bar* and *tpe* is an independent evolutionary branch (Chiang et al., 2012a).

Accordingly, we propose two hypotheses to explain the divergence of these two species with their high morphological similarity and distributional overlap. As the first hypothesis, we posit that these two species have been diverged for a long time, but similar selective pressures have led to their convergent morphologies (e.g., Yeaman et al., 2016). Their morphological differentiation is the relictual evidence of species divergence. In addition, its relatively small distributional range could also imply a bottleneck event for *tpe*. The second hypothesis is that the island endemic *tpe* is recently derived from the widespread species *bar*. This means that its relative small distributional range is the consequence of a founder event. The insufficient time for divergence has led to only slight morphological differences between them, except in certain minor characters, such as leaf shape and seed coat pattern (Huang et al., 2003).

In this study, the genetic variation and genetic differentiation between *bar* and *tpe* were assessed by multilocus markers and the plastid DNA sequences. We examined the genetic components for some small but stable morphological differences and the climatic niches occupied by these two species to assess the current stage of the species' divergence. The following speciation scenarios of island species were examined: early speciation with a subsequent bottleneck and secondary contact events, or recent founder speciation. In a further evolutionary scenario, tests were conducted to evaluate the single or multiple derivation of *tpe* in northern Taiwan, which provides an explanation for the phenomenon of interspecific gene flow and describes the process of genetic differentiation during the early stage of speciation (i.e., when the speciation process is not completed yet). In addition, the climate's effect on genetic divergence at the population level was further examined to understand the local adaption of these two allied species. Based on our genetic evidence, we suggest that the amount of genetic divergence between these two morphologically divergent organisms may not be sufficient to grant them the level of species.

## Materials and methods

### Current distribution and sampling

*Scutellaria barbata* is distributed in northern Taiwan. Some populations were recorded from western and northeastern Taiwan. This species is usually scattered in the sallow hills, on farmland ridges and along roadsides, and is usually distributed sporadically, making it hard to form a dense population. *Scutellaria taipeiensis* is very rare and restricted to the Taipei Basin of northern Taiwan. It grows attached to bare rock or the surrounding soil, usually in sunny environments along trails (Huang et al., 2003). Therefore, each population size of these two species is quite limited and uneven through their distribution range. We collected samples from populations of both *bar* and *tpe* distributed in the Taipei Basin, and the surrounding areas. One population of *bar* was sampled in southwestern Taiwan to test the effect of long-distance geographic and climatic differences. In total, six and three populations of *bar* and *tpe* were collected, respectively (Table 1). Because of their morphological similarity, we first sampled in the MK population, which is the type location of *tpe*; the collected samples were subsequently checked and confirmed by one of the original authors of *S. taipeiensis* (Mr. Arthur Hsiao). Identification of other *tpe* populations was based on the macromorphology and growth habit of the MK population. In total, 80 and 74 individuals of *bar* and *tpe* were sampled, respectively, for genotyping and sequencing. The seed coat patterns of all populations were further checked by Scanning Electron Microscopy (SEM).

**Table 1:** **The sampling sites and bioclimatic variables**.

**Species**	**Population**	**Abbreviation**	**Latitude**	**Longitude**	**Altitude (m)**	**bio2**	**bio8**	**bio9**	**bio13**	**bio18**	**bio19**
*S. barbata*	Yonghe	YH	25.00708	121.5280	15	6.04	27.73	17.58	300	782	376
	Caoling Historic Trail	CL	24.97636	121.9265	257	5.77	22.07	16.90	452	833	666
	Yilan River	YL	24.74310	121.7679	5	5.98	23.47	18.53	429	742	469
	Toucheng	TC	24.84704	121.7965	71	5.90	23.10	17.97	430	879	569
	Xinhua	XH	23.02808	120.3320	21	8.58	27.83	20.17	483	1,284	65
	Wulai	WL	24.86637	121.5498	474	6.18	23.27	14.12	452	1,179	495
*S. taipeiensis*	Maokong	MK	24.97265	121.5962	281	6.01	24.37	16.60	427	1,101	598
	Daan	DA	25.01841	121.5417	337	6.01	27.48	17.42	346	905	503
	Erge Mountain	EG	24.95750	121.6390	49	6.03	24.02	16.47	479	1,160	595

*bio2, mean of monthly temperature (max temp − min temp), or the mean diurnal range; bio8, mean temperature of the wettest quarter; bio9, mean temperature of the driest quarter; bio13, precipitation of the wettest month; bio18, precipitation of the warmest quarter; bio19, precipitation of the coldest quarter*.

### Scanning electron microscopy for seed coat pattern

Seeds were collected from all studied populations for SEM observation. An accelerating voltage of 15 kV was applied in order to obtain high image resolution signals. SEM was conducted using the tabletop SEM TM3000 (Hitachi, Tokyo, Japan).

### Sequencing and genotyping

Chloroplast DNA fragments *ndh*F-*rpl*32 and *rpl*32-*trn*L were chosen for sequencing. The PCR amplification and sequencing conditions followed Chiang et al.'s (2012a) protocol. According to Chiang et al.'s (2012b) primer test, 14 microsatellite primers were amplifiable for both *bar* and *tpe*. Herein these 14 primer sets were used to test for polymorphism at the population level; three monomorphic loci were discarded in the further analyses. The amplification and genotyping conditions followed Chiang et al.'s (2012b) protocol. All sequences were deposited in GenBank (Supplementary Table 1).

### Neutrality tests and genetic diversity

After sequence alignment and trimming the 5′ and 3′ ends to equal lengths, neutrality tests were conducted by Tajima's (1989) *D*-test with coalescent simulations. For the neutrality test of microsatellite loci, we used the Fdist (Beaumont and Nichols, 1996) and Bayesian-based outlier analysis of the multinomial-Dirichlet model (BayeScan analysis) to assess the *F*_ST_ distribution among all loci. The genetic diversity of chloroplast DNA sequences was then estimated by DnaSP 5.10 (Librado and Rozas, 2009). The genetic diversity of microsatellite loci was estimated using Arlequin 3.5 (Excoffier and Lischer, 2010) and GenAlEx 6.5 (Peakall and Smouse, 2012).

### Analysis of molecular variance

The analysis of molecular variance (AMOVA) was used for testing the degrees of genetic variation between species, among, and within populations. We conducted the AMOVA using Arlequin 3.5 (Excoffier and Lischer, 2010).

### Bayesian clustering analysis for examining the genetic components of populations

Bayesian clustering analysis (BCA) was used to examine the similarity and divergence of genetic components among populations. The BCA was performed using STRUCTURE 2.3.4 (Hubisz et al., 2009). A non-admixture model was applied to STRUCTURE with *a priori* sample localities. The posterior probability of grouping number (*K* = 1–10) was estimated by 10 independent runs using one-million steps Markov chain Monte Carlo (MCMC) replicates after a 100,000-step burn-in for each run to evaluate consistency. The best grouping number was evaluated by Δ*K* (Evanno et al., 2005) in STRUCTURE HARVESTER v. 0.6.94 (Earl and Vonholdt, 2012).

### Discriminant analysis of principal components for discriminating species and populations

The discriminant analysis of principal components (DAPC) was used for distinguishing *bar* and *tpe* and the populations that were subjectively identified. Properties of the “without *a priori*” using partial synthetic variables to minimize variation within groups (Manel et al., 2005; Jombart et al., 2010) could help to evaluate the artificial classification objectively. The DAPC was conducted using the R (R Core Team, 2015) package adegenet (Jombart, 2008). Kruskal–Wallis tests for the first two principal components (PCs) and the first two linear discriminants (LDs) of the DAPC were conducted to test the genetic divergence between species and between populations.

### Speciation scenarios for the approximate bayesian computation

**Figure 2:**
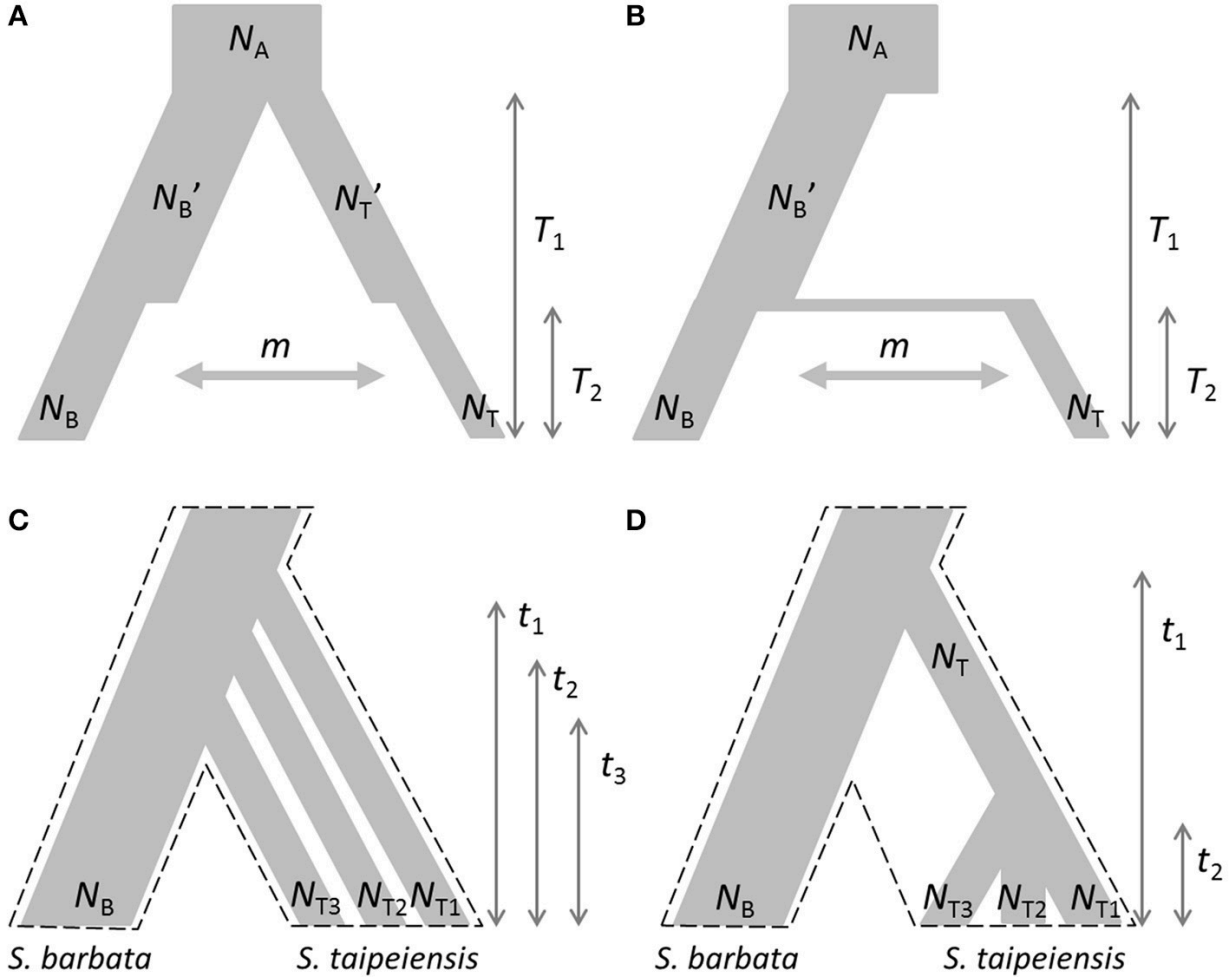
**Evolutionary scenarios of the divergence of ***S. barbata*** (***bar***) and ***S. taipeiensis*** (***tpe***) for approximate Bayesian computation (ABC)**. Contrasting scenarios illustrate **(A)** the bottleneck events after species divergence with recent gene flow (abbreviation: the secondary-contact scenario) and **(B)** the recent founder speciation of *tpe* derived from *bar* with continuous gene flow (abbreviation: the founder scenario). Two different contrasting extended scenarios of the founder scenario were further tested: **(C)** multiple origins of *tpe* from *bar* and **(D)** a single origin of *tpe* from *bar*. In scenarios **(C,D)**, migration rate was set as a free parameter and is not illustrated. The dashed frames in **(C,D)** indicate the pattern of species divergence.

Based on the estimated genetic variation and genetic structure and current geographic distributions, two evolutionary scenarios were proposed: the first is the demographic bottleneck of both *bar* and *tpe* under common environmental pressures with recent interspecific gene flow (secondary contact) after completed speciation (Figure 2A); the second scenario illustrates the recent founder speciation of *tpe* derived from the progenitor species *bar* with continuous gene flow (i.e., incomplete speciation, Figure 2B). We further tested two extended scenarios of the recent founder speciation scenario, namely that *tpe* originated multiple times (Figure 2C) or in a single origin from *bar* (Figure 2D). Testing these two extended scenarios helps clarifying whether the species-determining morphological traits are derived from the common ancestor or are the consequence of convergence. An approximate Bayesian computation (ABC) was used to test these evolutionary scenarios. First, package simcoal2 of the ABCtoolbox was used to generate one million pseudodata for simulation (Wegmann et al., 2010). We used arlsumstat to estimate diversity parameters (e.g., number of alleles, allele frequency, fixation indices *F*_ST_, *F*_IS_, *F*_IT_, etc.) of the pseudodata and transform them by the pls. To validate the best scenario, the general linear model (GLM) post-sampling regression adjustments on the best 5,000 simulated parameters were retained for obtaining the marginal density of models by the ABCestimator. The best evolutionary scenario was selected according to the Bayes factor and the ratio of marginal densities.

### Distinguishing the grinnellian niches (temperature and precipitations) between *bar* and *tpe*

Grinnellian niches are defined as the scenopoetic environmental variables a species requires to survive, such as the temperature and precipitation, etc. (Grinnell, 1917). Nineteen bioclimatic variables (Bioclim) that represent the Grinnellian niches were extracted from the WorldClim website (http://www.worldclim.org/bioclim). Principal component analysis (PCA) of independent climatic variables to reduce the dimensionality defining the niche space allowed comparison of the integrity of Grinnellian niches between *bar* and *tpe*. Firstly, the variables with a high variance inflation factor (VIF) were removed to reduce the multicollinearity. This procedure was repeated until the VIF values of all remaining variables were < 10 and finally six bioclimatic variables were retained for the further analyses. Species occurrence data were collected from the Global Biodiversity Information Facility (GBIF, http://www.gbif.org), herbarium records, public records available on the web, and our collecting sites. Unclear records and wrong identifications were ruled out. Finally, 56 records of *bar* and five records of *tpe* were used for the PCA. We further used multinomial logistic regression (MLGR) to test the effect of bioclimatic variables on the prediction of species occurrence. Because the simpler model of no correlation among predictors (bioclimatic variables) could not be rejected by the model of correlated predictors (likelihood ratio statistic = 12.214, df = 37, *P* = 0.99996), we used the model with independent predictors for MLGR analysis. The significance of each predictor was tested by the type-II analysis of variance (ANOVA) Wald χ^2^ test.

### Predicting potential distributions by ecological niche modeling

To predict the potential habitats of both *bar* and *tpe*, species distribution models were built under the maximum entropy model implemented in MaxEnt (Phillips and Dudík, 2008) and the R package dismo (Hijmans et al., 2013). The occurrence data of both species were obtained from our sampling sites, herbarium data, and GBIF. The bioclimatic variables were also downloaded from WorldClim with a resolution of 30 arc-sec (about 1 × 1 km). A maximum of 2,000 iterations of each species were conducted, and species occurrence data were randomly divided (10%) to train the model. One regularization multiplier and 10,000 background points were set to create models for each species set. The logistic output, consisting of a grid map with a suitability value range from 0 to 1, was generated and visualized with the R package maptools (Lewin-Koh et al., 2011).

### Mantel test for the isolation-by-distance and isolation-by-environment tests

To test how the geographic distance and environmental differences affect the genetic composition, the Mantel test of Spearman correlation was performed between genetic, geographic, and environmental (climatic) distances. Pairwise *F*_ST_ was calculated between populations based on Nei's (1977) approach and then Rousset's (1997) estimate *F*_ST_/(1 − *F*_ST_) was used as the genetic distance metric. Geographic distance was estimated using the Euclidean distance according to three dimensional factors (latitudes, longitudes, and altitudes). Environmental distance was also calculated using the Euclidean distance with six retained Grinnellian niches variables. A partial Mantel test between genetic and environmental distances controlled for the geographic distance was also done. A multiple matrix regression with randomization (MMRR) was further performed to test whether the genetic distance responds to the variation of geographic and/or environmental distances. We first examined the correlation between the genetic distance and geographic distance and between genetic distance and environmental distance. Autocorrelation between the geographic and environmental distances was further examined. Finally, the joint effect of both geographic and environmental distances on genetic distance was examined. Regression coefficients of Mantel test (ρ) and MMRR (β) and their significance were determined based on 9,999 random permutations.

### Testing the effect of climate on population genetic components

To understand whether the climatic variables (Grinnellian niches) explain the genetic distribution of populations of *bar* and *tpe*, partial distance-based redundancy analyses (partial dbRDA) were performed. The first five principal components (PC1–PC5) were used to represent the genetic components, and six retained climatic variables were used as predictors conditioning on the geographic distribution (latitude and longitude). Analysis of variance was further used to test the significance of each predictor. The partial dbRDA was conducted using the R package vegan. We further analyzed the distribution of climatic variables along ordination axes using the GLM. We also used the MLGR to test the effect of bioclimatic variables on populations. The type-II ANOVA Wald χ^2^ test was used to test the significance of each bioclimatic factor.

## Results

### Morphological similarity of the nutlet coat of mature seeds

**Figure 3:**
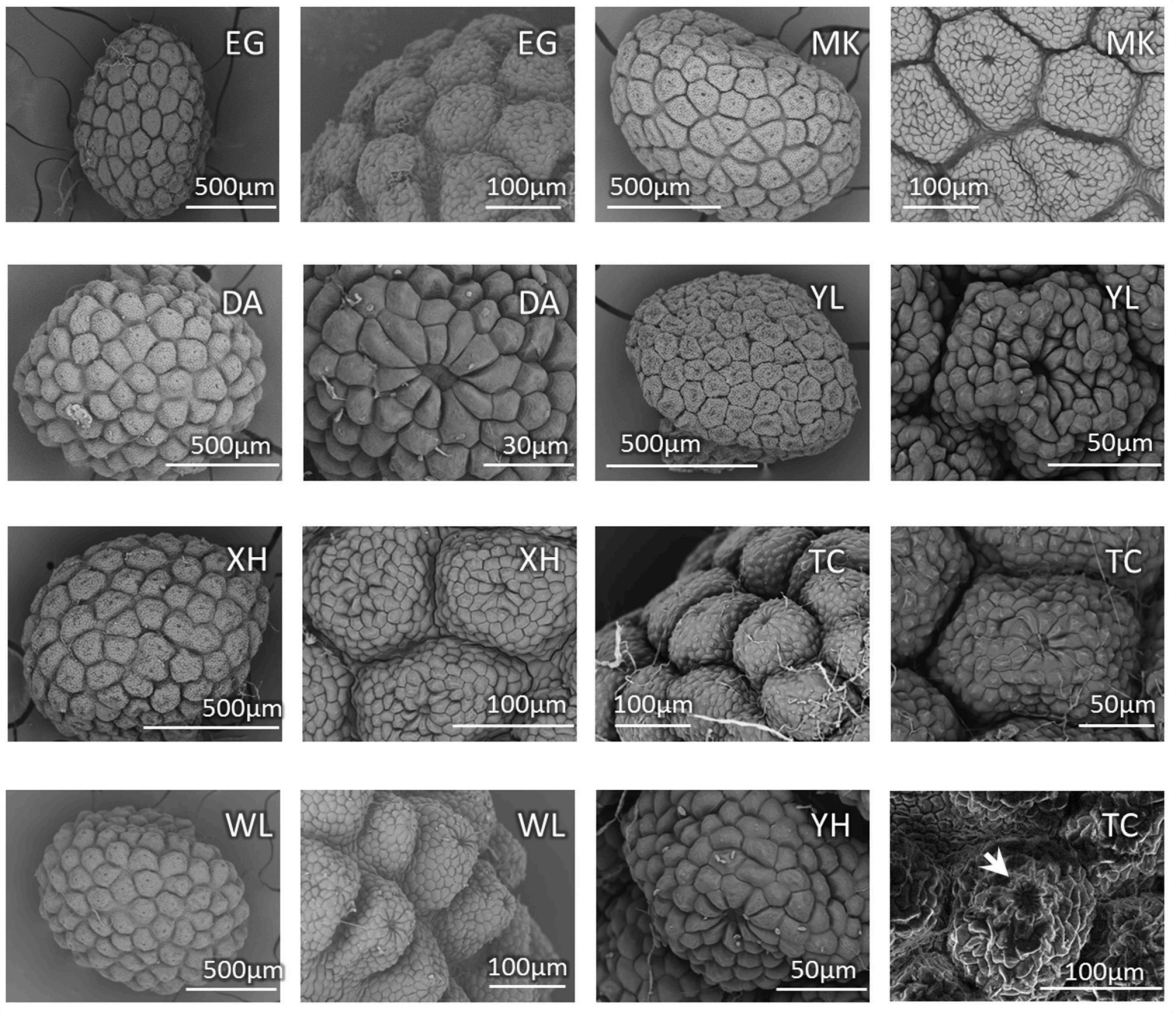
**Seed coat pattern of sampled populations (except CL) in this study**. The SEM pictures show similarities with slight size variations in the nutlet coats of mature seeds among populations but no obvious difference between species. The bottom-right picture shows the nutlet coat of an immature seed of population TC (*S. barbata*), in which the “radiated umbrella-like” structure can be observed (indicated by arrow). Population MK is the type location of *S. taipeiensis*.

Because the characters of vegetative morphology that are the most distinctive between *bar* and *tpe* are variable and can be affected by growth and nutrient conditions, we specifically checked the seed coat patterns, another distinguishing trait in the reproductive organs, to confirm the stability of this character for species delimitation. Based on our observations, there is slight variation in the seed size among individuals but no obvious difference between species (Figure 3). The SEM also shows that the nutlet coats of mature seeds in all populations of *bar* and *tpe* are of the rounded concentric type (i.e., type 3 described in Huang et al., 2003, Figure 3). The radiated umbrella-like nutlet coat (type 2) described by Hsieh and Huang (1995) was only seen in immature seeds (the bottom-left picture of Figure 3). After repeatedly comparing the nutlet-coat patterns, we believe that the differences between these two character states (i.e., rounded-concentric vs. radiated umbrella-like) reflect differences in seed maturity rather than differences between species.

### Neutrality tests and genetic diversity

Before estimating genetic diversity, we performed neutrality tests by Tajima's *D*-test for cpDNA sequences and by Fdist and the multinomial-Dirichlet model (BayeScan) for the outlier analysis for the microsatellite loci. No deviation from zero in Tajima's *D* (*D* = 1.315, *P* > 0.10, coalescent simulation *D* = −0.066, 95% confidence interval −1.550–2.112, *P* = 0.911) and no positive or negative outliers of microsatellite loci (Supplementary Figure 2) suggest that the genetic markers used in this study all evolve neutrally and are appropriate for further genetic population assessment and speciation model tests.

**Figure 4:**
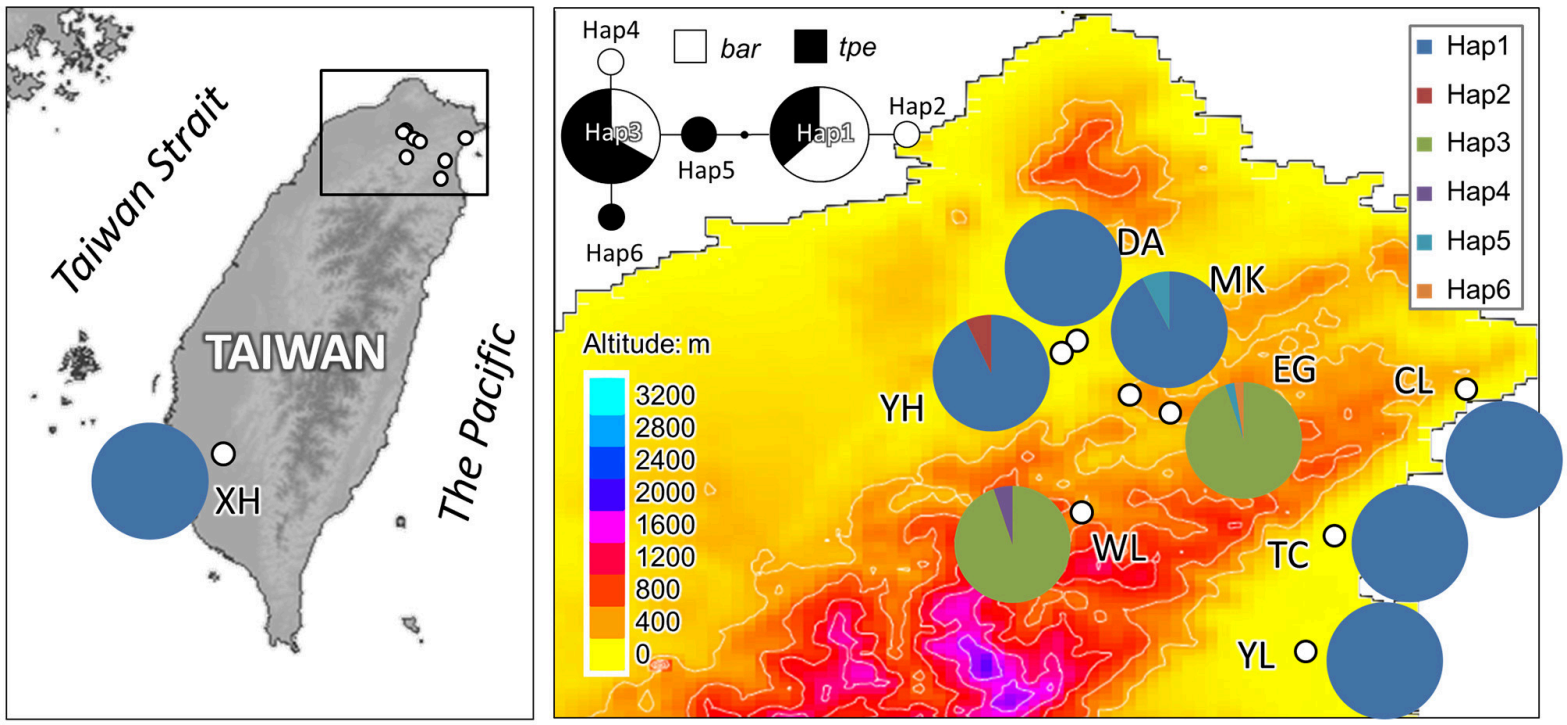
**Sampling sites and chloroplast haplotype distribution of ***S. barbata*** and ***S. taipeiensis*****. The minimum spanning network of haplotypes is also shown. Populations DA, MK, and EG are *S. taipeiensis*; populations XH, YH, WL, CL, TC, and YL are *S. barbata*.

In cpDNA, only the population YH of *bar* and populations MK and EG of *tpe* revealed nucleotide polymorphisms. The genetic diversity index Π of these three populations (YH, MK, and EG) is 0.143, 0.154, and 0.051, respectively, and the index θ_W_ is 0.314, 0.322, and 0.237, respectively. In total, when considering the indels six haplotypes were obtained, and the haplotype frequencies, distributions, and relationships (i.e., the minimum spanning tree) are illustrated in Figure 4. Genetic diversity assessed by microsatellite markers revealed varying degrees of genetic diversity among populations, ranging from 0.105 ± 0.039 to 0.296 ± 0.088 in expected heterozygosity (*H*_e_) in *bar* and from 0.189 ± 0.080 to 0.230 ± 0.069 in *H*_e_ in *tpe* (Table 2). Among the 11 loci examined, roughly half are polymorphic (5.556 ± 1.878 polymorphic loci) in every population and the high proportions of private alleles per locus (ranges from 0 to 0.364 ± 0.203, Table 2) indicate a genetic fixation phenomenon in small populations by the drift effect. The drift effect on small populations is reflected in the significantly high genetic differentiation among populations (*F* statistic = 0.681, *P* < 0.00001) with a relatively small proportion of genetic variation within populations (35.94% variation, *F* = 0.641, *P* < 0.00001; Table 3). However, genetic variation does not contribute to species divergence, i.e., genetic differentiation between species is lacking (*F* < 0, *P* = 0.820, Table 3).

**Table 2:** **Genetic diversity of the sampling populations estimated by cpDNA and microsatellite loci**.

**Statistics**	**YH**	**CL**	**YL**	**TC**	**XH**	**WL**	**MK**	**EG**	**DA**
**cpDNA**									
Sample size (*N*)	14	4	22	9	12	19	13	39	23
*S*	1	0	0	0	0	0	1	1	0
π	0.143	0	0	0	0	0	0.154	0.051	0
θ_W_	0.314	0	0	0	0	0	0.322	0.237	0
Tajima's *D* (*P*)	−1.155 (0.172)	0 (1)	0 (1)	0 (1)	0 (1)	0 (1)	−1.149 (0.168)	−1.126 (0.120)	0 (1)
**MICROSATELLITE DNA**									
Sample size (*N*)	12	4	22	9	12	21	12	40	23
No. polymorphic loci	5	4	9	5	5	5	3	8	6
No. different alleles	2.273 ± 0.407	1.545 ± 0.247	2.273 ± 0.237	1.545 ± 0.207	1.727 ± 0.304	1.818 ± 0.377	1.636 ± 0.310	2.545 ± 0.529	2.182 ± 0.615
No. effective alleles	1.748 ± 0.283	1.359 ± 0.170	1.161 ± 0.041	1.143 ± 0.057	1.440 ± 0.217	1.519 ± 0.248	1.410 ± 0.183	1.449 ± 0.177	1.368 ± 0.145
Shannon's index	0.515 ± 0.15	0.276 ± 0.120	0.266 ± 0.057	0.189 ± 0.071	0.321 ± 0.133	0.356 ± 0.147	0.304 ± 0.132	0.420 ± 0.130	0.346 ± 0.129
No. private alleles	0.273 ± 0.141	0.273 ± 0.195	0.182 ± 0.122	0.091 ± 0.091	0.364 ± 0.203	0.000 ± 0.000	0.091 ± 0.091	0.091 ± 0.091	0.091 ± 0.091
Expected heterozygosity (*H*_e_)	0.296 ± 0.088	0.173 ± 0.075	0.128 ± 0.029	0.105 ± 0.039	0.191 ± 0.079	0.212 ± 0.084	0.189 ± 0.080	0.230 ± 0.069	0.197 ± 0.069

**Table 3:** **Analysis of molecular variance (AMOVA) of populations of two ***Scutellaria*** species in Taiwan in microsatellite loci**.

**Source of variation**	**df**	**Sum of squares**	**Variance components**	**% variation**	***F*** **statistic**	***P***
Among species	1	51.794	−0.391	−12.72	−0.127	0.820
Among populations	7	518.882	2.359	76.78	0.681	<0.00001
Within populations	301	332.398	1.104	35.94	0.641	<0.00001
Total	309	903.074	3.073			

### Population structure of *bar* and *tpe*

**Figure 5:**
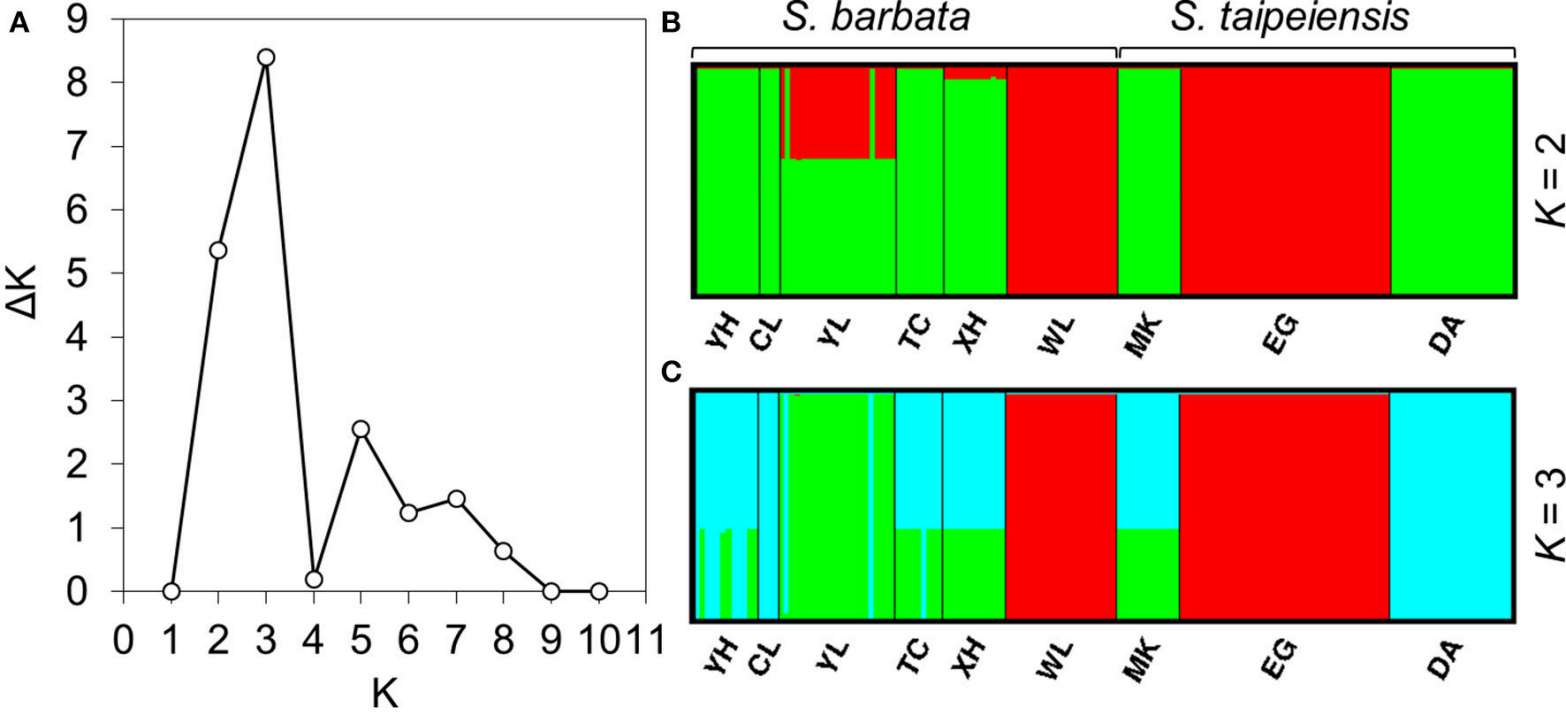
**Results of the Bayesian clustering analysis conducted using STRUCTURE. (A)** The Δ*K* plot shows that *K* = 3 gets the highest Δ*K* value and *K* = 2 gets the second-best Δ*K* value, meaning that the most probable grouping number could be three or two. The clustering patterns of genetic components by **(B)** two groups, **(C)** three groups.

To understand the pattern of genetic distribution of these two species, we performed a BCA in STRUCTURE and ordination analysis by DAPC. The STRUCTURE result suggested the best grouping number (*K*) is 3 and the second-best *K* is 2 based on the Δ*K* (Figure 5A). When *K* = 2, population EG of *tpe* and population WL of *bar* were inferred to be composed of identical genetic components, and population YL of *bar* is composed of both genetic components (Figure 5B). When *K* = 3, DA of *tpe* and YL of *bar* were inferred to be two further components different from EG and WL, and the other populations were comprised of both genetic components of DA and YL (Figure 5C). These two inferences are similar and consistently indicate that the populations WL and EG are divergent from the other populations, and such divergence is inconsistent with the species delimitation.

**Figure 6:**
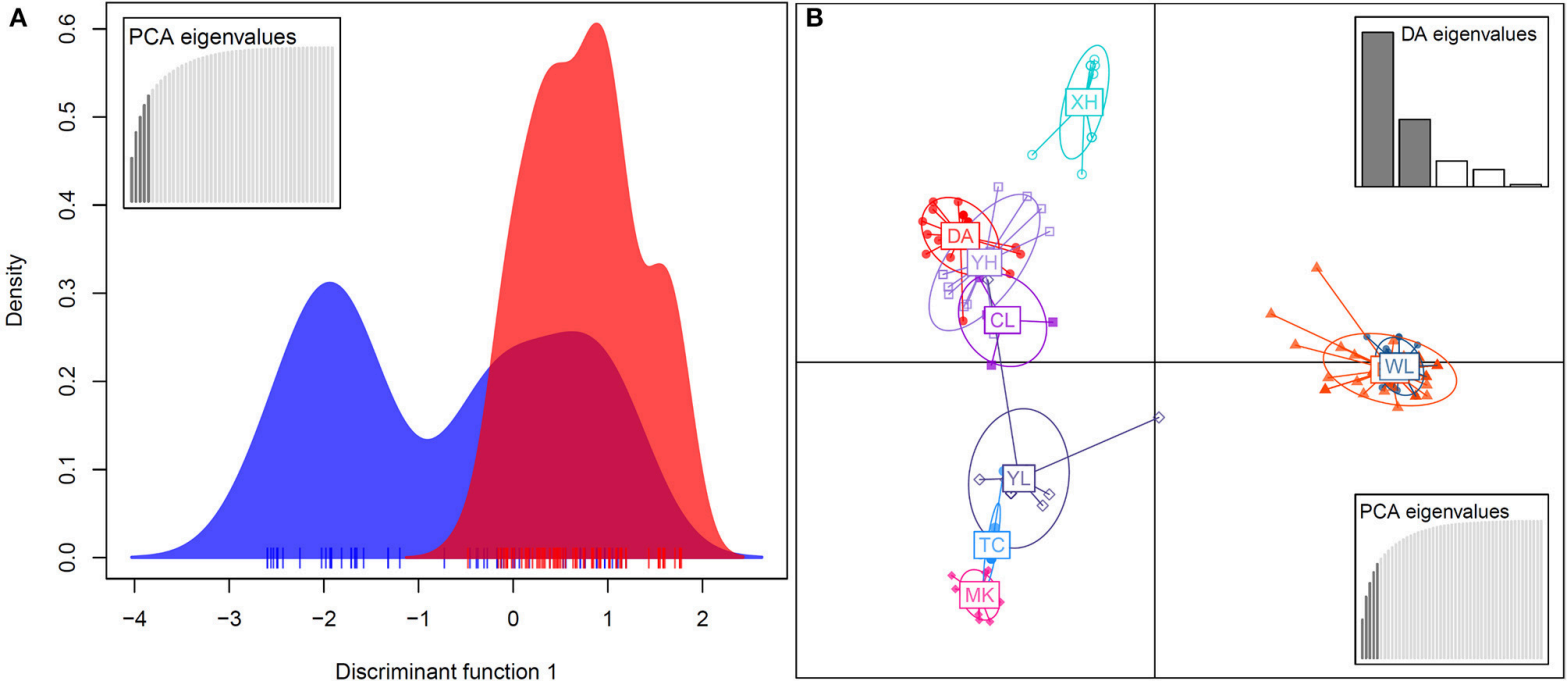
**Population structure revealed by the discriminant analysis of principal components (DAPC). (A)** The DAPC for separating the species *S. barbata* (*bar*, blue) and *S. taipeiensis* (*tpe*, red). **(B)** The DAPC for separating populations of *bar* and *tpe*. The red series belongs to *tpe* and the blue-color series are *bar*.

In the DAPC analysis, which uses one discriminant function to distinguish five principal components (PCs), a broader range of genetic variation was detected in *bar* than in *tpe*, and the range of genetic variation of *tpe* is skewed but within the range of *bar* at the species level (Figure 6A). A further DAPC analysis on populations shows a similar result, i.e., that the range of genetic variation of three populations of *tpe* was clustered within the range of *bar*. Two main clusters were distinguished by two discriminant functions for five PCs: one is the cluster of the WL of *bar* and EG of *tpe*, the other populations belonging to another cluster (Figure 6B). The clustering pattern inferred by STRUCTURE and DAPC is congruent.

**Figure 7:**
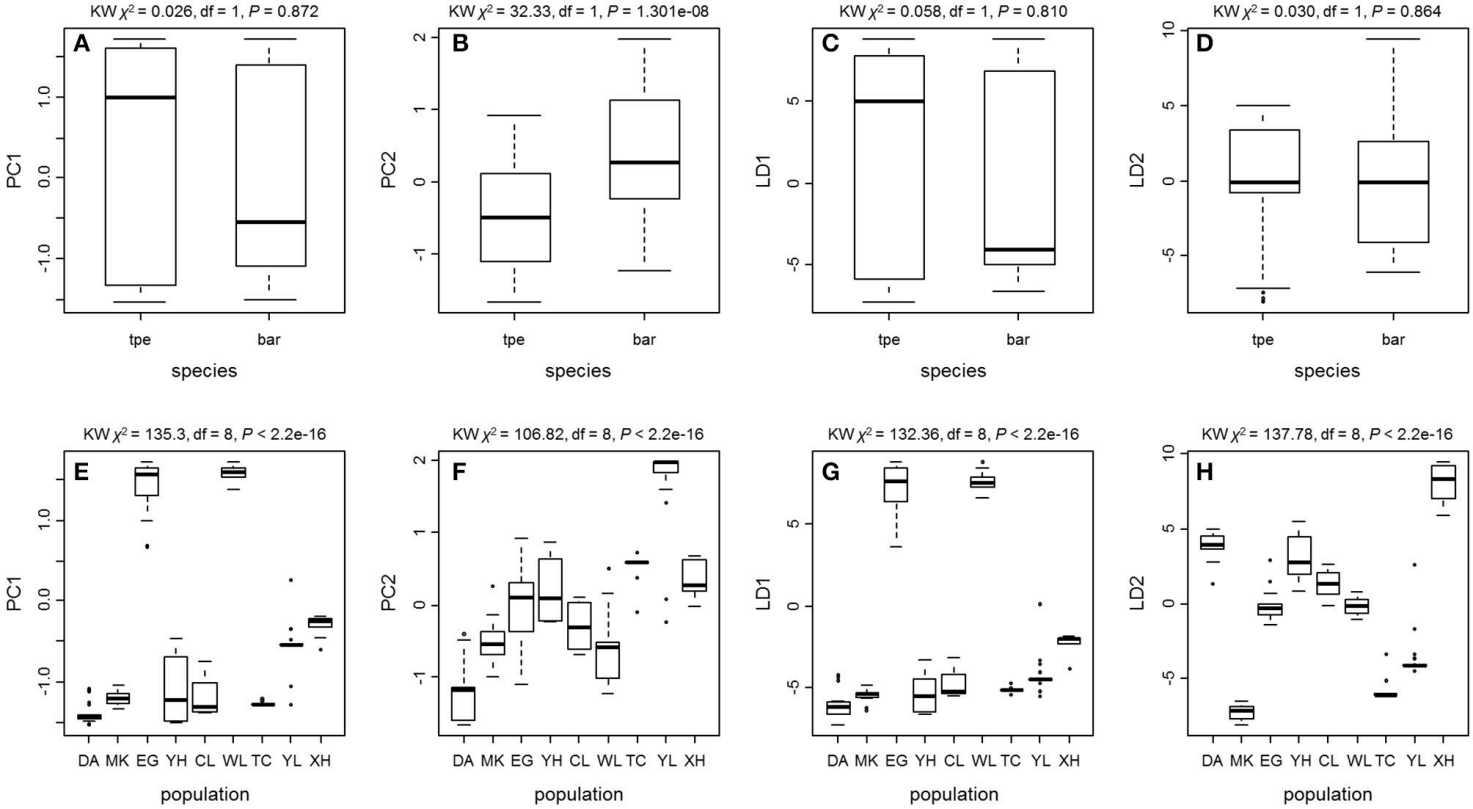
**The Kruskal–Wallis test of the first two principal components and the first two linear discriminants of the genetic variation at microsatellite loci reveals no or little genetic divergence between species but significant population differentiation. (A–D)** Comparisons between species. **(E–H)** Comparisons among populations.

We further tested the differences in genetic variation according to the transformed variables of the first two principal components PC1 and PC2 of PCA and the first two linear discriminants LD1 and LD2 of DAPC at both the species level and the population level. Only the transformed variable PC2 (Kruskal–Wallis test, χ^2^ = 32.33, df = 1, *P* = 1.301e–08) revealed species divergence between *bar* and *tpe* but no species-level differences were found in PC1 (*P* = 0.872), LD1 (*P* = 0.810), or LD2 (*P* = 0.864; Figure 7). At the population level, all four transformed vectors showed significant differences in genetic variation among populations (Kruskal–Wallis test, *P* < 2.2e–16, Figure 7). Taken together, these results suggest obvious population divergence but no or less genetic differentiation between species.

### Multiple origins with very recent divergence explain the current situation of no genetic differentiation between species

**Figure 8:**
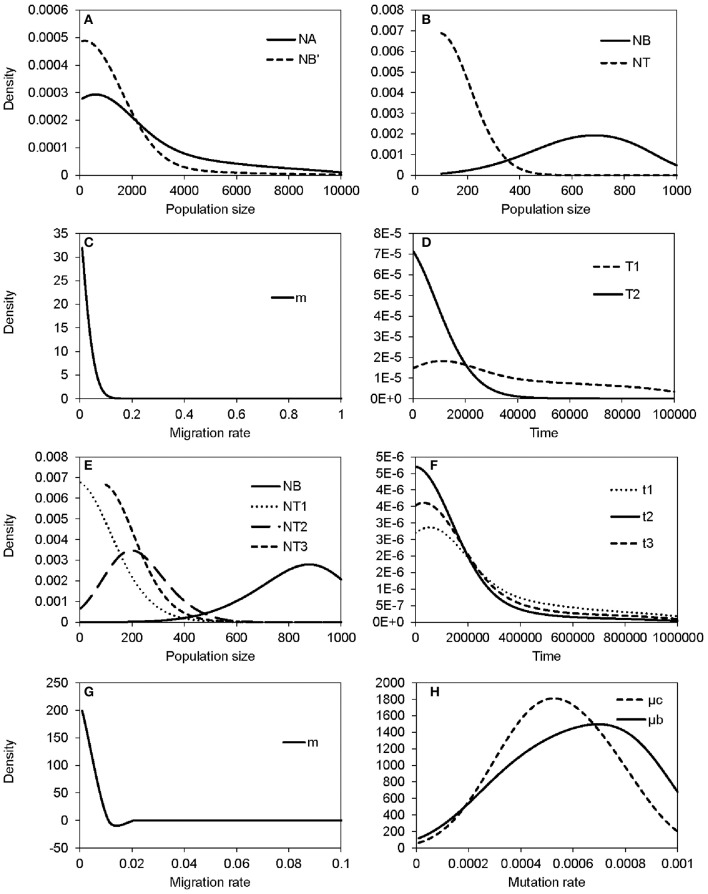
**Summary results of the ABC analyses. (A–D)** display the distribution of estimated parameters of Figure 2B: **(A)** The estimated population sizes of ancestral populations *N*_A_ and *N*_B′_; **(B)** the estimated population sizes of current populations *N*_B_ and *N*_T_; **(C)** the migration rate; **(D)** the coalescent time of *S. barbata* (*bar*, T_1_) and the time of founder speciation of *S. taipeiensis* (*tpe*, T_2_). **(E–G)** display the distribution of estimated parameters of Figure 2C: **(E)** The population sizes of *bar* (*N*_B_) and three populations of *tpe* (*N*_T1_–*N*_T3_); (f) colonization times of three populations of *tpe*; **(G)** the migration rate; **(H)** the distribution of the estimated mutation rates of scenario Figure 2B (μb) and Figure 2C (μc).

The non-significant genetic differentiation between species may suggest frequent gene flow geographically after secondary contact, or alternatively, the sharing of large amounts of ancestral polymorphism. Following these two possibilities, evolutionary scenarios of early divergence with secondary contact (Figure 2A) and recent founder speciation with incomplete divergence (Figure 2B) were tested using ABC. The former scenario (secondary contact) scored a marginal density of 9.24 × 10^−13^ (0.199%), much lower than the founder-speciation scenario (marginal density: 4.63 × 10^−10^, 99.801%). The estimated ancestral population size of *bar* is suggested to have decreased under the founder-speciation scenario (*N*_A_ = 597.487, 95% CI: 164.557–8172.980; *N*_B_' = 199.497, 95% CI: 126.479–4701.65, Figure 8A). However, a recovery of the current population size was estimated (*N*_B_ = 683.417, 95% CI: 235.953–958.165), but the founder populations of *tpe* have a very small effective population size (*N*_T_ < 100, Figure 8B). The estimated time since the founder speciation of *tpe* is less than 100 generations (Figure 8D), indicating the poor inference of species divergence despite no or little interspecific gene flow (*m* < 0.01, Figure 8C) between *bar* and *tpe*. The estimated mutation rate (μ) of each locus per generation in secondary contact scenario has been estimated (μb = 6.965 × 10^−4^, 95% CI: 2.238-9.703 × 10^−4^, Figure 8H).

Since the divergence time is extremely short, we wondered whether *tpe* is a good species or not. If it is, populations of *tpe* are expected to be a monophyletic group. Two evolutionary scenarios were further tested following the founder speciation scenario: multiple origins (i.e., polyphyly) of *tpe* (Figure 2C) and a single origin followed by divergence (i.e., monophyly) in *tpe* (Figure 2D). The multiple origins scenario has a higher marginal density (5.577 × 10^−49^, 99.836%) than the single origin (marginal density: 9.167 × 10^−52^, 0.164%), indicating a rejection of the monophyly of *tpe*. Similar to the estimation above, the effective population size of populations of *tpe* is smaller than that of *bar* (Figure 8E), while the divergence times have wide ranges from < 100 generations to 55,370 generations (95% CI: 6,814–861,759 generations; Figure 8F). The estimated mutation rate (μ) of each locus in multiple origins scenario has also been estimated (μc: 5.274 × 10^−4^, 95% CI: 1.999-8.9 × 10^−4^, Figure 8H). Such results of a wide range of divergence times of *tpe* populations from *bar* reflect (1) recent divergence and (2) sharing common ancestral polymorphisms that results in long coalescence times. This result also suggests that the divergence time is insufficient for complete speciation. Therefore, populations of both *bar* and *tpe* are combined into a single taxonomic group for the further analysis of local climatic adaptation. However, the very small migration rate on average (*m* < 0.001, Figure 8G) indicates that the gene flow seems to have ceased after population colonization.

### The overlapping grinnellian niches in *bar* and *tpe*

After removing 13 bioclimative variables of collinearity, the remaining six bioclimatic variables are: bio2 [mean of monthly (max temp − min temp), named the Mean Diurnal Range], bio8 (mean temperature of wettest quarter), bio9 (mean temperature of driest quarter), bio13 (precipitation of wettest month), bio18 (precipitation of warmest quarter), bio19 (precipitation of coldest quarter). Among these six bioclimatic variables, the first three (bio2, bio8, and bio9) belong to the temperature dimension, while the last three (bio13, bio18, and bio19) are the precipitation dimension.

**Figure 9:**
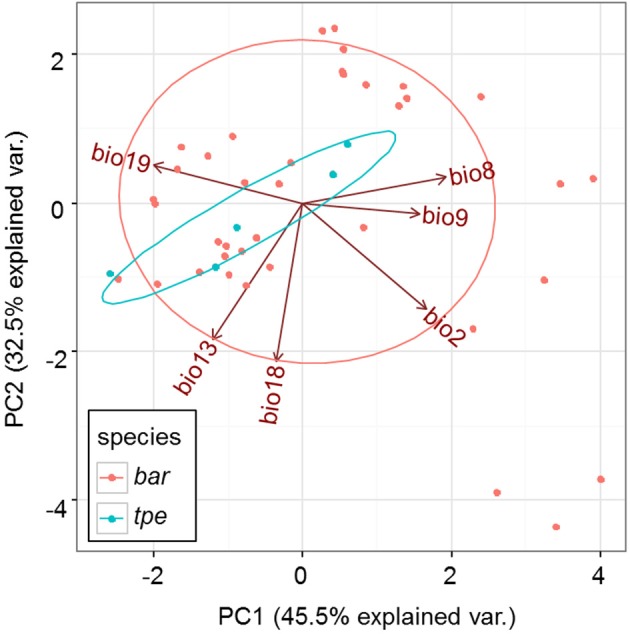
**Principal component analysis of the climate factors of ***S. barbata*** and ***S. taipeiensis*****.

Although multicollinearity was prevented by removing variables with VIF > 10, certain variables were still partially intercorrelated. For example, the temperature variable bio2 was negatively correlated with the precipitation variable bio19, and two precipitation variables, bio13 and bio18, are negatively correlated (Supplementary Figure 3). The partial correlation of bioclimatic variables shown by PCA also revealed that the explanatory direction for the Grinnellian niche distribution is opposite for bio2 and bio19, and that bio13 and bio18 have similar explanatory intensity and directions (Figure 9). The PCA plot drawn on the first two axes explains 45.5 and 32.5% of the variation in the Grinnellian niche, but this biaxial PC distribution cannot separate *bar* and *tpe* well (Figure 9). When solely considering the temperature dimension (bio2, bio8, and bio9), or solely considering the precipitation dimension (bio13, bio18, and bio19), similar results of indistinguishable clustering between *bar* and *tpe* were revealed (Supplementary Figure 4). It is worth noting that the explanatory percentage of the first two PCs of the precipitation dimension (95.2%, Supplementary Figure 4B) was estimated to be higher than that of the temperature dimension (84.2%, Supplementary Figure 4A), suggesting that the precipitation could be more relevant in explaining the geographic distribution of these two species. Among these six non-collinear bioclimatic variables, only bio8 (mean temperature of wettest quarter) was shown by the MLGR analysis to be significant for predicting the occurrence of species (*P* = 0.036, Supplementary Table 2).

### Spatial distribution predicted by ecological niche modeling

Ecological niche modeling (ENM) was further used to predict the suitable distributions of both *bar* and *tpe* as well as to examine the key climatic variables in the prediction. Distribution models of both species showed high discrimination performance. The cross-validation area under the curve (AUC) value for all models is 0.999, indicating that 99.9% of the records were correctly predicted. The climatic suitability of each species is proportional to similar climatic variables. Bio18 (precipitation of warmest quarter) and bio2 (mean diurnal range) are the first two contributors to the species distribution model in *bar* (57.9 and 28%, respectively) and in *tpe* (27.7 and 47.6%, respectively).

The ranges of both species predicted by the six bioclimatic variables (bio2, bio3, bio8, bio13, bio18, and bio19) are roughly consistent with the currently known distributions, except for a certain probability (< 50%) of suitable habitats predicted in the Hengchun Peninsula of southern Taiwan for the *tpe* (Supplementary Figure 1). In accordance with the specimen records, northern Taiwan is suggested as the most suitable habitat for both species by ENM. In contrast to the smaller ranges of *tpe*, the predicted distribution of *bar* is broader, including the most southern area and the northeastern and northwestern areas of Taiwan (Figure 1 and Supplementary Figure 1). The central area of Taiwan, where the Central Mountain Range is, is not suitable for either species distribution.

### Fitness of isolation-by-distance and isolation-by-environment models

To assess whether the geographic or the environmental difference drives the genetic divergence among populations, isolation-by-distance and isolation-by-environment tests were conducted using the Mantel test. The Spearman correlation shows no significant correlation between geographic and environmental distances (ρ = 0.2888, *P* = 0.0872), between genetic and geographic distances (ρ = 0.2454, *P* = 0.0659), or between genetic and environmental distances (ρ = 0.1105, *P* = 0.3606; Table 4). The partial Mantel test did not detect significant correlation between genetic and environmental distance when conditioning on the geographic effect either (ρ = 0.2743, *P* = 0.0814, Table 4). Similar results of no autocorrelation between geographic and environmental distances (*r*^2^ = 0.0152, β = 0.1201, *P* = 0.501) and non-significant effects of geographic (*r*^2^ = 0.0373, β = 0.1418, *P* = 0.281) and environmental distances (*r*^2^ = 0.0063, β = 0.0600, *P* = 0.685) on the change of genetic distance were obtained by MMRR analyses (Table 4). The joint effect of both geographic and environmental distances also did not affect the genetic distance significantly (*r*^2^ = 0.0404, β_geo_ = 0.1367, *P* = 0.345, β_env_ = 0.0426, *P* = 0.797, Table 4). These results indicate that the population genetic differentiation (or gene flow) is not linearly correlated with the geographic and climatic differentiation.

**Table 4:** **Summary of the Mantel test and multiple matrix regression with randomization (MMRR) between the genetic (gen), geographic (geo), and environmental (env) distances**.

	**Mantel test**		**MMRR**		
	**ρ**	***P***	***r*****^2^**	**β**	***P***
geo vs. env	0.2888	0.0872	0.0152	0.1201	0.501
gen vs. geo	0.2454	0.0659	0.0373	0.1418	0.281
gen vs. env	0.1105	0.3606	0.0063	0.06	0.685
gen vs. env|geo^a^	0.2743	0.0814	–	–	–
(gen vs. env × geo)^b^	–	–	0.0404	β_geo_: 0.1367	0.345
				β_env_: 0.0426	0.797

a *The partial Mantel test*.

b *The joint effect of both environmental and geographic distances in MMRR*.

### Explanation of the population genetic variations by environmental factors

Despite nonlinear correlation between the climatic differentiation and genetic distance, we still wondered whether the local climatic heterogeneity explains the genetic variation of populations. When conditioning on the geographic distribution, we found that 59.5% of the variation is explained by climatic variables (Table 5). The ANOVA further indicates that all six predictors (bio2, bio8, bio9, bio13, bio18, and bio19) can significantly explain population genetic components (*P* < 0.0001, Table 5), and the bio9 and bio2 have the highest explanatory proportions (23.0 and 11.7%, respectively) for predicting the population genetic variation (Table 5). The distribution of climatic variables along the ordination axis was further examined by GLM. Four bioclimatic variables including two variables (bio8 and bio9) of the temperature dimension and two variables (bio13 and bio18) of the precipitation dimension have significant *F* statistics (adjusted *R*^2^ = 0.041, 0.104, 0.216, and 0.178, *P* < 0.05, Table 5), but only the precipitation variables bio13 and bio18 correlate significantly with the ordination axis1 of dbRDA, while all four variables are significantly correlated with axis2 (Table 5). However, the adjusted *R*^2^ of bio8 could be too small (roughly explaining 4% of the variation on axis2) to be meaningful. If only the geographic clusters are considered (i.e., locations of populations) without genetic information, all the bioclimatic variables cannot predict the occurrence of populations by MLGR independently. Nevertheless, the joint effect of the four factors, bio8 × bio13 × bio18 × bio19, can predict the populations by MLGR significantly (χ^2^ = 26.540, df = 8, *P* = 0.0008).

**Table 5:** **Summary of partial dbRDA, showing the significance of climatic variables (constrained factors) for explaining the variation in the genetic components**.

					**GLM for the distr. of climatic variables along ordination axes**						
	**Inertia (Var)^a^**	**Proportion**	***F***	**Pr (>***F***)**	***t*** **(axis 1)**	**Pr (>|***t***|)**	***t*** **(axis 2)**	**Pr (>|***t***|)**	**Adj ***R***^2^**	***F***	***P***
Conditional	1.000	0.251									
Unconstrained (Residual)	0.614	0.154									
Constrained	2.367	0.595									
bio2	0.466	0.117	110.753	<0.0001	−0.171	0.865	−0.347	0.729	−0.012	0.076	0.927
bio8	0.318	0.080	75.602	<0.0001	1.347	0.180	2.568	0.011	0.041	4.294	0.015
bio9	0.917	0.230	218.132	<0.0001	−1.290	0.199	4.296	3.09E-05	0.104	9.928	8.86E-05
bio13	0.323	0.081	76.837	<0.0001	−3.347	0.001	−5.669	7.03E-08	0.216	22.150	3.61E-09
bio18	0.197	0.049	46.852	<0.0001	−3.040	0.003	−5.034	1.34E-06	0.178	17.680	1.25E-07
bio19	0.147	0.037	35.062	<0.0001	−1.802	0.074	−1.462	0.146	0.022	2.759	0.067
Total	3.982	1.000									

a *Inertia is the mean squared Euclidean distance*.

## Discussion

### Indistinguishable morphological characters

*Scutellaria taipeiensis* is a species morphologically alike to *bar*. It was named in 2003 based on its leaf shape (length-to-width ratio < 2 and triangular-ovate to broadly ovate shape) and the rounded concentric tuberculae type of nutlet coat that distinguished it from *bar* (Huang et al., 2003). However, in our SEM observation, we found that the nutlet coat pattern is not different between species and the “radiated umbrella-like” character (Hsieh and Huang, 1995) may disappear when seeds mature (Figure 3). In other words, the morphological differences of nutlet-coat patterns described by Hsieh and Huang (1995) and Huang et al. (2003) may result from the comparison of seeds at different developmental stages. In addition, the leaf size and shape is usually plastic in response to environmental changes (Rozendaal et al., 2006; Royer et al., 2009), and the range of leaf shape is wider and more varied in *bar*, covering the range of *tpe* (Supplementary Table 3). Morphological similarity in both vegetative and reproductive organs implies that *tpe* could merely be an ecotype or form of *bar* instead of a distinct species. To confirm this speculation, a comprehensive study of the genetic and ecological properties was carried out to complement the morphological evidence (Camargo et al., 2012).

### Lack of genetic and climatic niche differentiation between species implies an incomplete speciation process

The genetic variation of *tpe* is essentially a subset of that found in *bar* (Figure 6A), which matches the geographic distribution of these two species (Figure 1 and Supplementary Figure 1). In addition, both genetic and niche components are non-significantly different between species (Figures 7A–D, 9, Supplementary Figure 4). These analyses suggest that the speciation is incomplete, or at least that species divergence is not reflected in the overall genetic differentiation and niche preference. Our result resembles the common phenomenon that the progenitor species is paraphyletic to its derived species in plants because the ongoing gene flow increases the time for the derived species to achieve monophyly (Rieseberg and Brouillet, 1994). However, in this case, the daughter species *tpe* does not form a monophyletic group but fits the model of multiple founder speciation events (Figure 2B).

In fact, the estimated time of founder speciation of *tpe* is quite short (< 100 generations) in contrast to the long coalescent time of the Taiwanese populations of *bar* (11,145 generations ago, 95% CI: 1,505–92,795 generations ago, Figure 8D). If a generation is taken as 1 year, the coalescent time of the Taiwanese population of *bar* is roughly at the end of the Last Glacial Maximum (LGM), the time that Taiwan Island was most recently connected to continental Asia (Nino and Emery, 1961; Voris, 2000), which is probably the time when *bar* colonized Taiwan. However, the extremely short divergence time of *tpe* supports the hypothesis that *tpe* is not a good species but could be a subset of *bar*, or alternatively, that the loci that contribute to the population's differentiation are not involved in the species' divergence (Table 3). Shared polymorphisms across closely related species appear to be common in island species (Lopez-Sepulveda et al., 2013; Takayama et al., 2015), which might result from introgression following secondary contact (Minder and Widmer, 2008) or from maintaining ancestral polymorphisms due to incomplete lineage sorting within recently diverged species (Nagl et al., 1998). The ABC analysis suggested that sharing ancestral polymorphism could better explain the genetic structure of *bar* and *tpe* than secondary contact did. Furthermore, the best-fit scenario of multiple origins suggests insufficient time for lineage sorting in neutral genes after the divergence of speciation genes or speciation traits (i.e., the genes or traits involved in speciation). Multiple origins from a single progenitor usually are seen as an evolutionary mechanism for the recent and rapid radiation of island species rather than the mature radiation that sometimes leads to a high diversity of continental species (cf. Linder, 2008). However, such an evolutionary mechanism of multiple origins could be unstable to fusion and convergence to monophyly.

### Climatic variables are not associated with species delimitation

The Grinnellian niche that is comprised of temperature and precipitation is important and related to plant speciation, in particular in those budding speciation cases with peripheral ranges or distributional overlaps (Martinez-Cabrera et al., 2012; Grossenbacher et al., 2014). Although no positively selected loci was detected (Supplementary Figure 2), variation of neutral genes could still be affected due to demographic changes (e.g., population size decline, Figures 8A,B) under strong environmental pressures (Schoville et al., 2012). Bio8 is the only bioclimatic variable that has a significant effect on the prediction of groups of species. Bio8 is the mean temperature of the wettest quarter. However, a non-significant difference in bio8 was found between *bar* (24.72 ± 2.273°C) and *tpe* (24.66 ± 2.947°C) in means (independent two-group Mann–Whitney *U*-test, *W* = 132.5, *P* = 0.854) and variance (Kruskal–Wallis test, χ^2^ = 0.039, df = 1, *P* = 0.844). This is probably because the overlapping distribution ranges of *bar* populations and *tpe* populations result in a similar climatic situation, thus causing a false positive for species prediction by the climate factor.

Despite the lack of species divergence in genetics and niches, local climatic heterogeneity has driven the genetic differentiation among populations within the island. This phenomenon is very like the case of *Aeonium davidbramwelii* and *A. nobile* on the island of La Palma in the Canarian archipelago: the former is an ecological generalist while the latter is an ecological specialist. There is a lack of species divergence but an obvious population structure exists (Harter et al., 2015). However, in our case, although the niche space of *tpe* is smaller than that of *bar*, their spatial distribution predicted by ENM on the basis of six bioclimatic variables is almost overlapping (Supplementary Figure 1). The only certain difference with the real distribution of *tpe* in the Taipei Basin is that the spatial model predicts a moderate probability for the potential distribution of *tpe* in southern Taiwan (Supplementary Figure 1). The dispersal mechanism of *Scutellaria* species is suggested to be attributable to rivers (Williams, 1992; Middleton, 2000; Chiang et al., 2012a). The lack of rivers running north to south in Taiwan decreases the probability of a southward long-distance dispersal of *tpe*.

### Climate heterogeneity leads to local adaptation

Considering all genetic, evolutionary, ENM, and morphological evidence, we conclude that no species delimitation can be made between *bar* and *tpe*. Therefore, all populations are taken as one species. A climatic effect, in particular the precipitation dimension, leads to local population differentiation. Although, geographic and environmental distances do not matter in the genetic differentiation among populations (Table 4), we still propose that the climatic variables determine the genetic components of populations inferred by both partial dbRDA (Table 5) and MLGR significantly. These results suggest that climatic heterogeneity is not linearly correlated with the genetic composition, but could affect genetic structure independently and locally, i.e., climate leading to local adaptation.

The environmental heterogeneity, which causes significant genetic divergence (Figures 7E–H) and contributes a high proportion of population genetic variation (76.78%, Table 3), could be a major driver leading to postzygotic isolation (Martin and Willis, 2007; Anacker and Strauss, 2014). According to the AMOVA, roughly 3/4 of the variation can be attributed to population divergence (Table 3), and the partial dbRDA indicates that ~60% of the variation among populations can be explained by constrained variables (i.e., climatic factors, Table 5). This means that roughly half the genetic variation can be attributed to local climatic heterogeneity. Environmental heterogeneity could increase the genetic or genomic barriers among populations, and thus result in local adaption (Kawecki and Ebert, 2004; Savolainen et al., 2007).

### Precipitation from late summer to early autumn is the key climatic factor responsible for the current genetic distribution

In the analyses that did not take the genetics into account, the joint effect of bio8 × bio13 × bio18 × bio19 inferred by MLGR and of bio2/bio18 inferred by ENM are suggested as the main climatic variables affecting population clustering and distribution; if the genetic factor is considered, the bio13/bio18 on axis1 and bio9/bio13/bio18 on axis2 of the dbRDA are suggested as significant explanations for the genetic variation among populations. The climatic variables bio18 and bio13 are two key variables inferred from different statistical analyses (except bio13 by the ENM). These two variables belong to the precipitation dimension, standing for the precipitation of the warmest quarter and the precipitation of the wettest month, respectively (Supplementary Table 4). The significant effect of bio13 and bio18 was revealed in the multiple hills (clusters) of the ordisurf plots of bio13 and bio18 in partial dbRDA (Supplementary Figures 5D,E) in contrast to the other bioclimatic factors (Supplementary Figures 5A–C,F), and their high correlation is also revealed in the almost overlapping coordinate axes (i.e., arrows) of the original bioclimatic variables bio13 and bio18 (Supplementary Figure 5).

According to the WorldClim database, the wettest month is September (340.4 mm on average) and the warmest quarter of all recorded populations is July to September (29.63°C on average; Supplementary Figure 6). In fact, bio13 and bio18 are highly correlated (Spearman's rank correlation, ρ = 0.757, *P* = 1.75e–12). According to the common characteristics of bio13 and bio18 as well as their significant correlation, we suggest that the main determinant governing the genetic structure of populations is actually the precipitation from late summer to early autumn. This period is during the typhoon season (late summer and early autumn) in Taiwan and just after the flowering and fruiting season (*tpe*: Mar–Jun; *bar*: Oct–May; Hsieh and Huang, 1995; Huang et al., 2003) with subsequent germination and growth. This is the most important period for population regeneration in this group of species (i.e., *bar* and *tpe*). Precipitation during seed germination and growth could affect the demographic size and could also influence the success of seed colonization. In addition, since the seed dispersal of *Scutellaria* species is thought to be spread by water (Williams, 1992; Cruzan, 2001), the abundant rainfall in typhoon season may also contribute to the long-distance dispersal, and further affects the population genetic structure.

## Conclusions

Spatial climatic heterogeneity is usually suggested as an important driver leading to population differentiation, even accelerating speciation. Taxonomic splitters sometimes name a species for slight morphological differences and link these characters to conjectural environmental differences. Here we provide integrated evidence including morphology, genetics, climatic niche modeling, and evolutionary tests to validate the taxonomic treatment of *S. taipeiensis*. When all these aspects are considered, all sampled populations should be regarded as the same species, i.e., *S. barbata*. We further find that the precipitation in the typhoon season is an important climatic agent that influences the population genetic structure significantly. Such periodic episodes of climatic events rather than the long-term constant climatic variation explain the nonlinear relationship between genetic and climatic differences. By taking into account the small-scale spatial effect of climate heterogeneity and its impact on genetic diversity, our study indicates the importance of local climatic episodes in governing the genetic diversity of plants.

## Author contributions

PL conceived the study; HH, BH, and JC conducted the molecular experiments. YH sampled and identified species. HH and BH analyzed the data. PL wrote the paper. HH, BH, CH, and PL critically reviewed and edited the manuscript. All authors have read and approved the final manuscript.

## Funding

This research was financially supported by the Ministry of Science and Technology (MOST) in Taiwan (MOST 102-2621-B-003-005-MY3) and also subsidized by the National Taiwan Normal University (NTNU), Taiwan.

### Conflict of interest statement

The authors declare that the research was conducted in the absence of any commercial or financial relationships that could be construed as a potential conflict of interest.

## Supplementary material

Supplementary Table 1 **Accession number of each haplotype**.

Supplementary Table 2 **Multinomial logistic regression analysis and the significance test for the bioclimatic effect on predicting species cluster by type-II ANOVA**.

Supplementary Table 3 **Leaf shape of ***Scutellaria barbata*** and ***S. taipeiensis*****.

Supplementary Table 4 **Codes for the bioclimatic variables used in this study**.

Supplementary Figure 1 **The predicted spatial distribution of two ***Scutellaria*** species in Taiwan based on six bioclimatic variables (bio2, bio3, bio8, bio13, bio18, and bio19) in (A)** *S. barbata* and **(B)** *S. taipeiensis*. Crosses on the maps are the distribution of specimen records.

Supplementary Figure 2 **Neutrality tests for examining the ***F***_**ST**_ distribution of 11 microsatellite loci by (A)** the Fdist approach and **(B)** the BayeScan approach. Both analyses show that none of the examined loci have extremely high (positive outlier) or low (negative outlier) *F*_ST_, suggesting all loci used in identifying species *bar* and *tpe* are neutral.

Supplementary Figure 3 **Correlation of six climatic variables**. The upper and lower panels are the magnitude of the correlation and scatterplots with the confidence ellipse and smoothed line, respectively. Blue and red colors encode the sign of positive and negative correlation, respectively. bio2, mean of monthly (max temp − min temp), or the mean diurnal range; bio8, mean temperature of wettest quarter; bio9: mean temperature of driest quarter; bio13, precipitation of wettest month; bio18, precipitation of warmest quarter; bio19, precipitation of coldest quarter.

Supplementary Figure 4 **Principal component analysis of (A)** the temperature factors (bio2, bio8, and bio9) and **(B)** the precipitation factors (bio13, bio18, and bio19) for *S. barbata* and *S. taipeiensis*. Bioclimatic variables were extracted from the WorldClim website (http://www.worldclim.org/bioclim).

Supplementary Figure 5 **Scatter and ordisurf plots of the partial dbRDA for six bioclimatic variables. (A)** bio2, **(B)** bio8, **(C)** bio9, **(D)** bio13, **(E)** bio18, **(F)** bio19.

Supplementary Figure 6 **The average temperature of every quarter (three months) (A)** and the average precipitation of every month **(B)** of the records of the distribution of *bar* and *tpe*.
