# Supplementary material for: Local Climate Heterogeneity Shapes Population Genetic Structure of Two Undifferentiated Insular Scutellaria Species
Source: Front Plant Sci. 2017 Feb 10;8:159. doi: 10.3389/fpls.2017.00159 (PMC5301026; doi:10.3389/fpls.2017.00159)
Supplement: Supplementary Table 1 — Accession number of each haplotype. [file Table1.DOCX]

**Supplementary Table S1.** Accession number of each haplotype.

|  | ndhF-rpl32 | rpl32-trnL |
| --- | --- | --- |
| Haplotype 1 | KY458956 | KY458962 |
| Haplotype 2 | KY458957 | KY458963 |
| Haplotype 3 | KY458958 | KY458964 |
| Haplotype 4 | KY458959 | KY458965 |
| Haplotype 5 | KY458960 | KY458966 |
| Haplotype 6 | KY458961 | KY458967 |
