# Supplementary material for: Local Climate Heterogeneity Shapes Population Genetic Structure of Two Undifferentiated Insular Scutellaria Species
Source: Front Plant Sci. 2017 Feb 10;8:159. doi: 10.3389/fpls.2017.00159 (PMC5301026; doi:10.3389/fpls.2017.00159)
Supplement: Supplementary Table 2 — Multinomial logistic regression analysis and the significance test for the bioclimatic effect on predicting species cluster by type-II ANOVA. [file Table2.DOCX]

**Supplementary Table S2.** Multinomial logistic regression analysis and the significance test for the bioclimatic effect on predicting species cluster by type-II ANOVA.

| Variable | Values | Std. Err. | LR χ^2^ | df | Pr(>χ^2^) |
| --- | --- | --- | --- | --- | --- |
| Intercept | 36.988 | 0.003 |  |  |  |
| bio2 | -20.704 | 0.052 | 2.622 | 1 | 0.105 |
| bio8 | 1.460 | 0.417 | 4.385 | 1 | 0.036* |
| bio9 | 1.577 | 0.682 | 0.905 | 1 | 0.341 |
| bio13 | 0.029 | 0.017 | 2.305 | 1 | 0.129 |
| bio18 | 0.013 | 0.006 | 0.941 | 1 | 0.332 |
| bio19 | -0.005 | 0.005 | 0.226 | 1 | 0.635 |

* Pr < 0.05.
