# Supplementary material for: Local Climate Heterogeneity Shapes Population Genetic Structure of Two Undifferentiated Insular Scutellaria Species
Source: Front Plant Sci. 2017 Feb 10;8:159. doi: 10.3389/fpls.2017.00159 (PMC5301026; doi:10.3389/fpls.2017.00159)
Supplement: Supplementary Table 3 — Leaf shape of Scutellaria barbata and S. taipeiensis. [file Table3.DOCX]

**Supplementary Table S3.** Leaf shape of *Scutellaria barbata* and *S. taipeiensis*.

| Species | Shape | Length (cm) | Width (cm) | Reference | URL |
| --- | --- | --- | --- | --- | --- |
| *S. taipeiensis* | triangular-ovate or broadly ovate | 0.8–1.5 | 0.5–1.2 | Huang et al. (2003) |  |
| *S. barbata* | triangular-ovate to ovate-lanceolate | 1.3–3.2 | 0.5–1.4 | eFlora of China; Flora of China | http://www.efloras.org/florataxon.aspx?flora_id=2&taxon_id=200020287; http://frps.eflora.cn/frps/Scutellaria%20barbata |
|  | narrowly ovate to ovate | 1.0–3.0 | 0.5–1.0 | Hsieh and Huang (1995); Flora of Taiwan | http://tai2.ntu.edu.tw/ebook/ebookpage.php?volume=4&book=Fl.%20Taiwan%202nd%20edit.&page=532 |
|  | - | 3 | 1 | India Biodiversity Protal | http://indiabiodiversity.org/species/show/263476 |
|  | lance-shaped or triangular | <3 | - | eFlora of India; GBIF | http://www.gbif.org/species/113619033 |
