# Supplementary material for: Local Climate Heterogeneity Shapes Population Genetic Structure of Two Undifferentiated Insular Scutellaria Species
Source: Front Plant Sci. 2017 Feb 10;8:159. doi: 10.3389/fpls.2017.00159 (PMC5301026; doi:10.3389/fpls.2017.00159)
Supplement: Supplementary Table 4 — Codes for the bioclimatic variables used in this study. [file Table4.DOCX]

**Supplementary Table S4.** Codes for the bioclimatic variables used in this study.

| Code | Bioclimatic variables |
| --- | --- |
| bio1 | Annual Mean Temperature |
| bio2 | Mean Diurnal Range (Mean of monthly (max temp - min temp)) |
| bio3 | Isothermality (BIO2/BIO7) (* 100) |
| bio4 | Temperature Seasonality (standard deviation *100) |
| bio5 | Max Temperature of Warmest Month |
| bio6 | Min Temperature of Coldest Month |
| bio7 | Temperature Annual Range (BIO5-BIO6) |
| bio8 | Mean Temperature of Wettest Quarter |
| bio9 | Mean Temperature of Driest Quarter |
| bio10 | Mean Temperature of Warmest Quarter |
| bio11 | Mean Temperature of Coldest Quarter |
| bio12 | Annual Precipitation |
| bio13 | Precipitation of Wettest Month |
| bio14 | Precipitation of Driest Month |
| bio15 | Precipitation Seasonality (Coefficient of Variation) |
| bio16 | Precipitation of Wettest Quarter |
| bio17 | Precipitation of Driest Quarter |
| bio18 | Precipitation of Warmest Quarter |
| bio19 | Precipitation of Coldest Quarter |
