# Supplementary figures and images for: Local Climate Heterogeneity Shapes Population Genetic Structure of Two Undifferentiated Insular Scutellaria Species
Source: Front Plant Sci. 2017 Feb 10;8:159. doi: 10.3389/fpls.2017.00159 (PMC5301026; doi:10.3389/fpls.2017.00159)

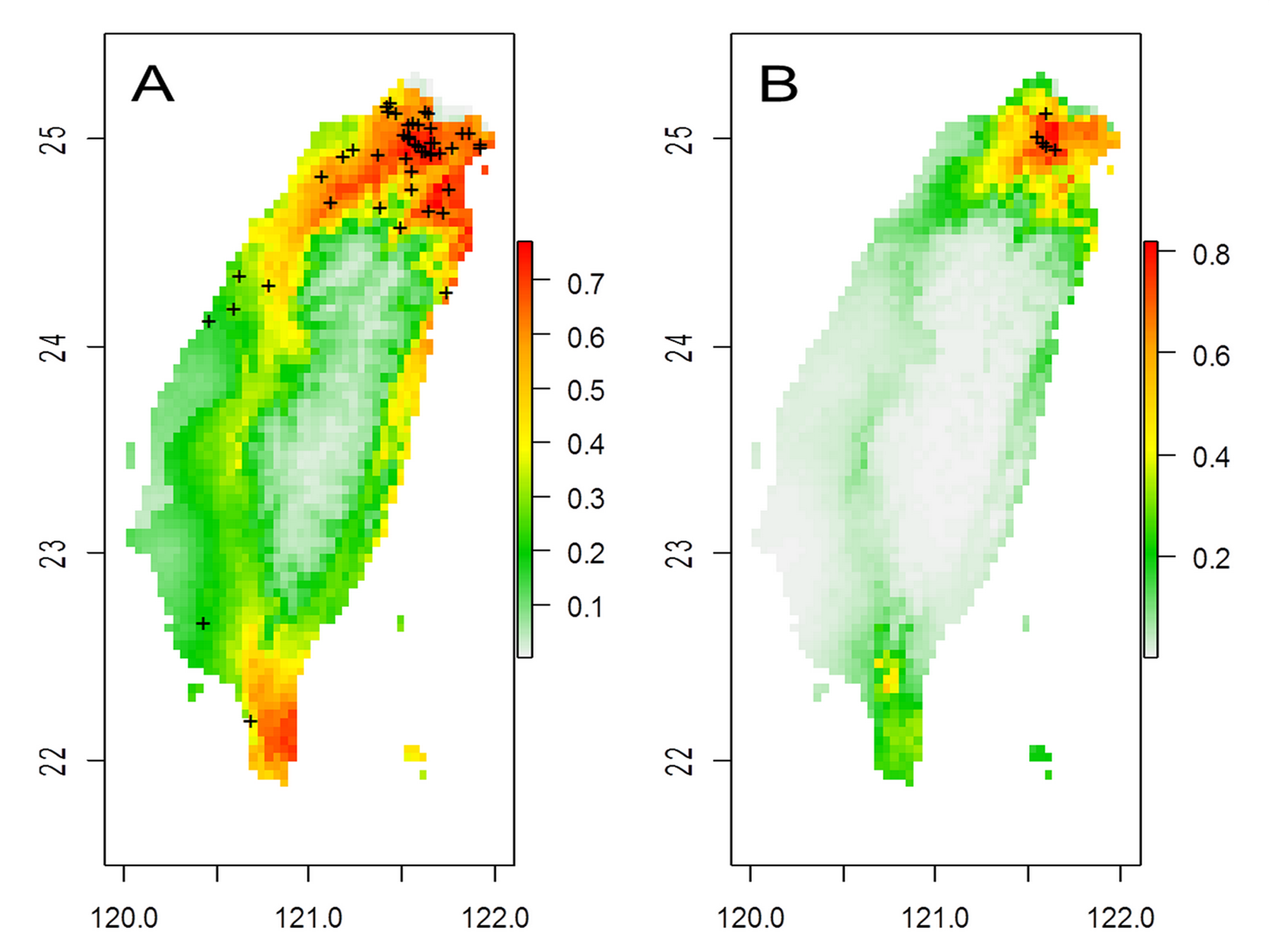

Supplement: Supplementary Figure 1 — The predicted spatial distribution of two Scutellaria species in Taiwan based on six bioclimatic variables (bio2, bio3, bio8, bio13, bio18, and bio19) in (A) S. barbata and (B) S. taipeiensis. Crosses on the maps are the distribution of specimen records. [file Image1.TIF]

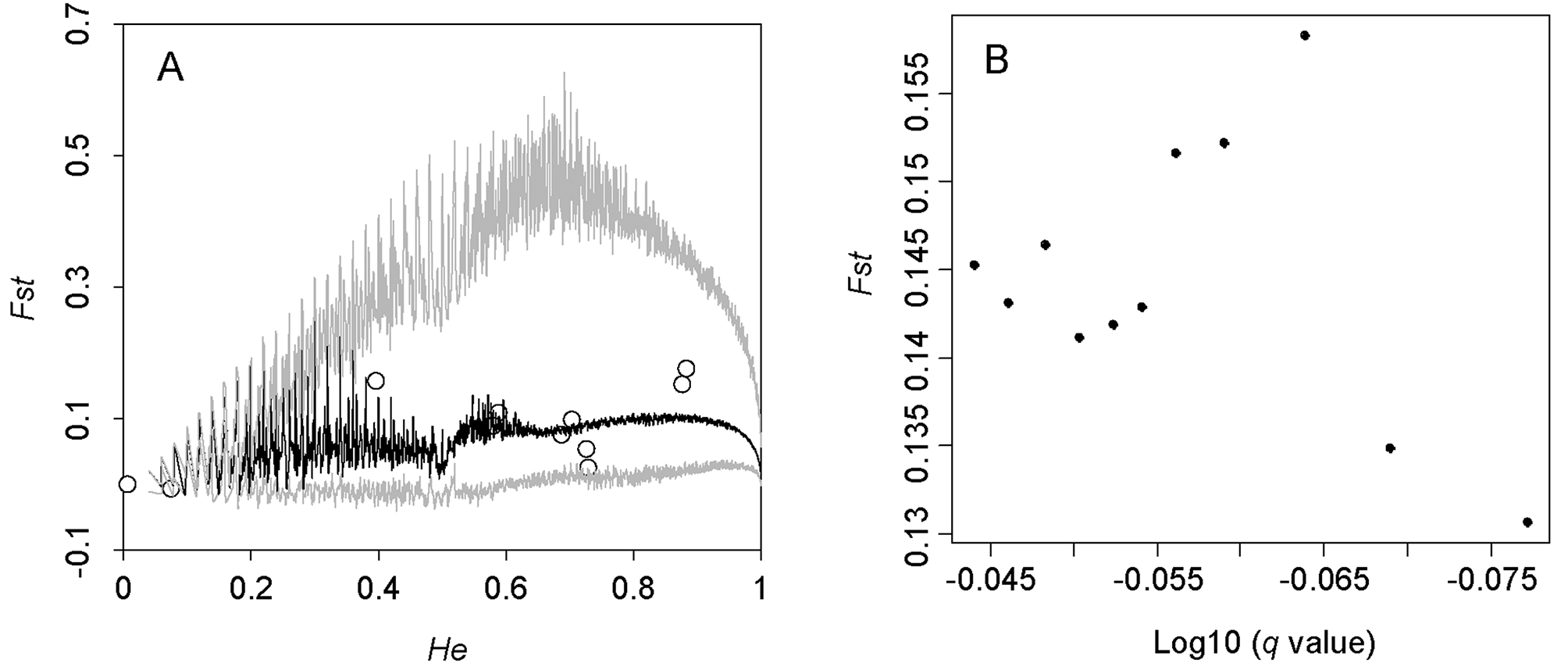

Supplement: Supplementary Figure 2 — Neutrality tests for examining the FST distribution of 11 microsatellite loci by (A) the Fdist approach and (B) the BayeScan approach. Both analyses show that none of the examined loci have extremely high (positive outlier) or low (negative outlier) FST, suggesting all loci used in identifying species bar and tpe are neutral. [file Image2.TIF]

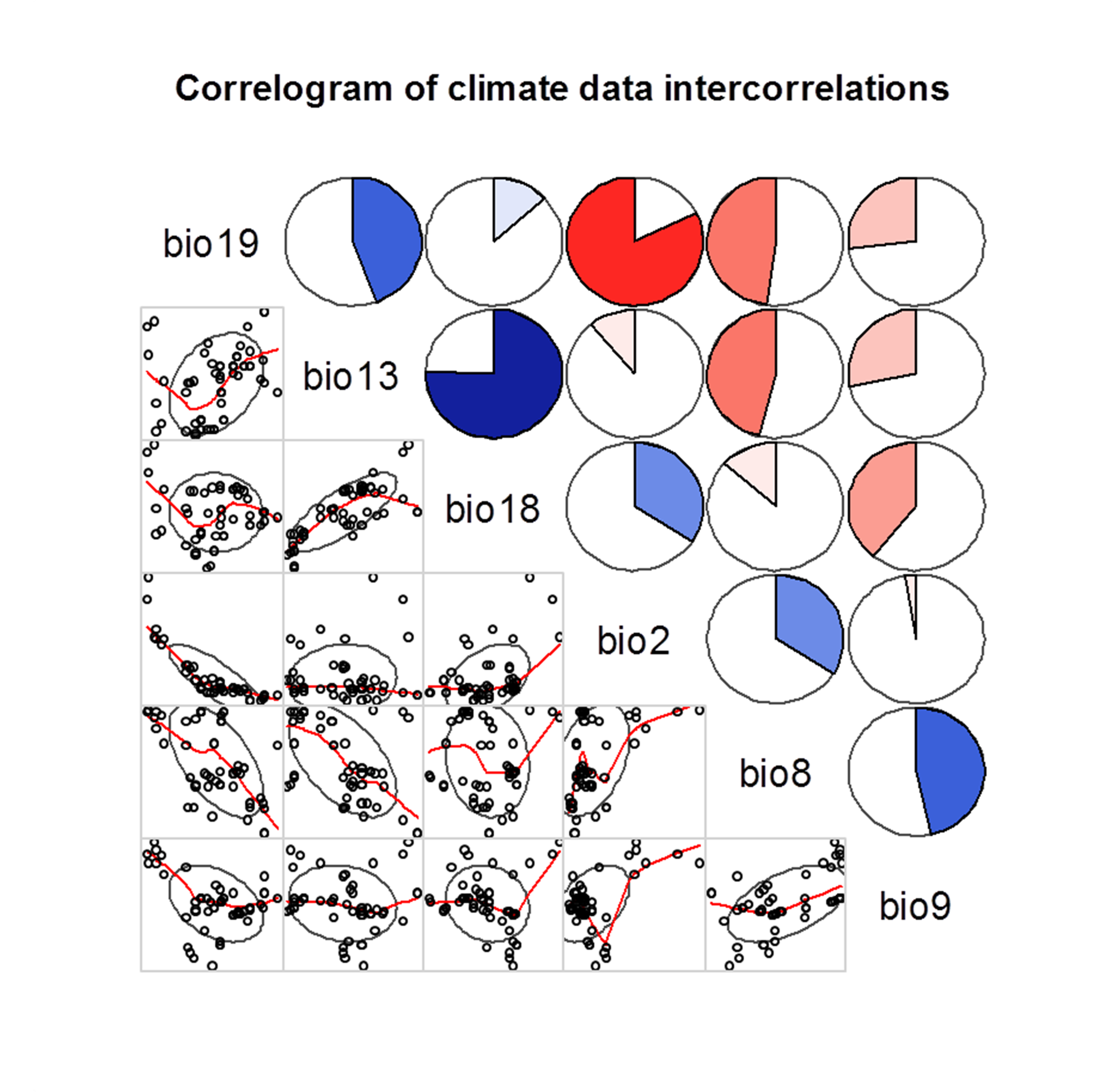

Supplement: Supplementary Figure 3 — Correlation of six climatic variables. The upper and lower panels are the magnitude of the correlation and scatterplots with the confidence ellipse and smoothed line, respectively. Blue and red colors encode the sign of positive and negative correlation, respectively. bio2, mean of monthly (max temp − min temp), or the mean diurnal range; bio8, mean temperature of wettest quarter; bio9: mean temperature of driest quarter; bio13, precipitation of wettest month; bio18, precipitation of warmest quarter; bio19, precipitation of coldest quarter. [file Image3.TIF]

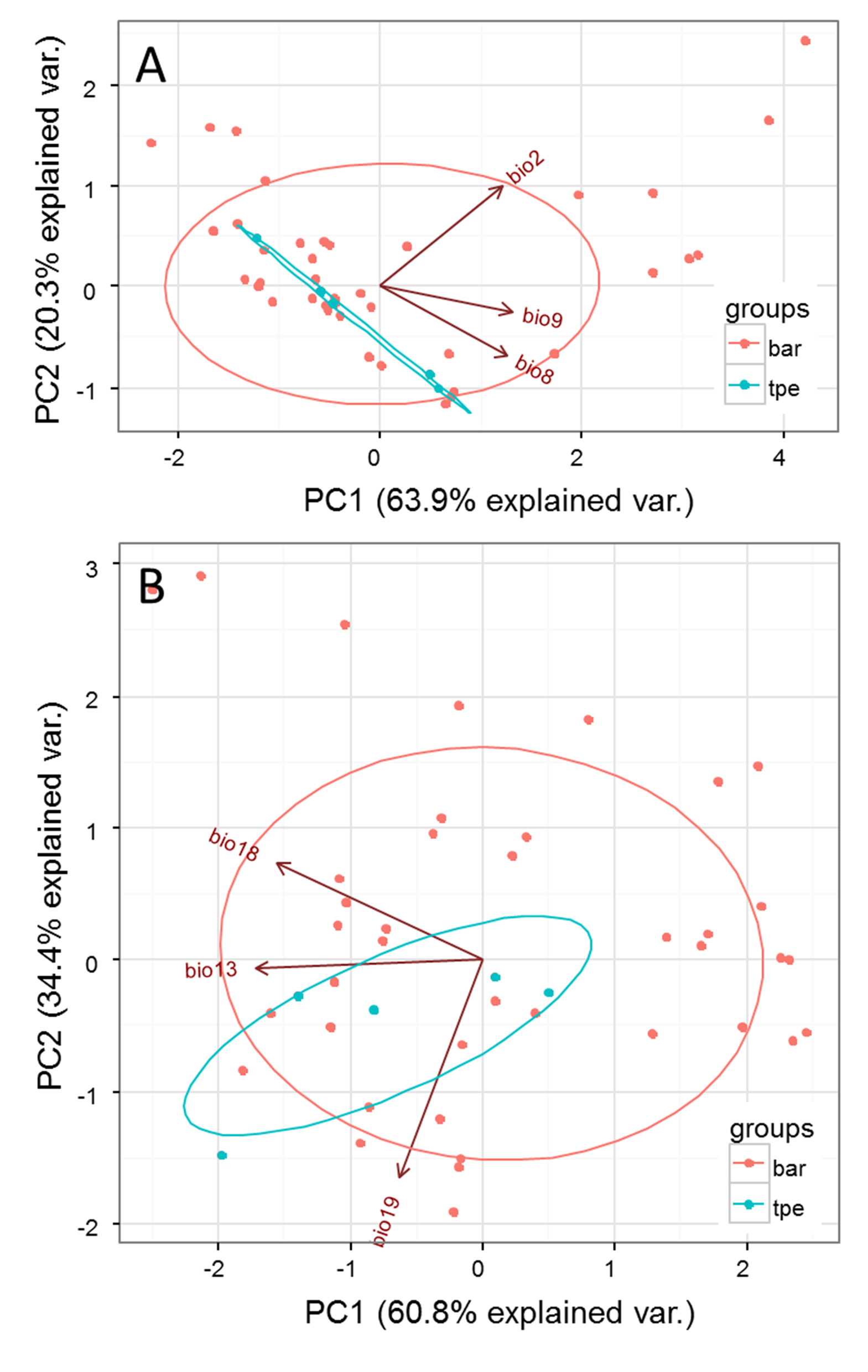

Supplement: Supplementary Figure 4 — Principal component analysis of (A) the temperature factors (bio2, bio8, and bio9) and (B) the precipitation factors (bio13, bio18, and bio19) for S. barbata and S. taipeiensis. Bioclimatic variables were extracted from the WorldClim website (http://www.worldclim.org/bioclim). [file Image4.TIF]

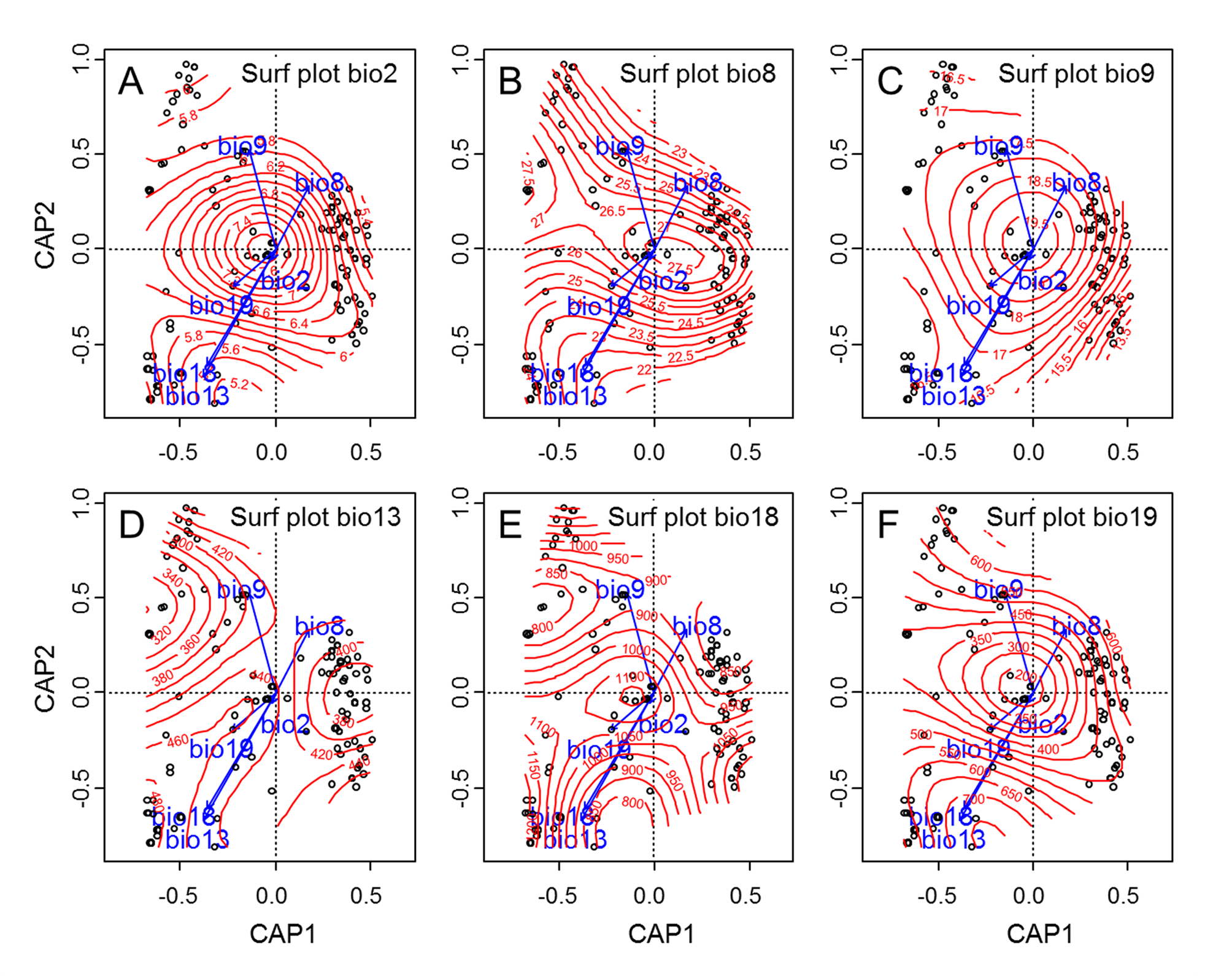

Supplement: Supplementary Figure 5 — Scatter and ordisurf plots of the partial dbRDA for six bioclimatic variables. (A) bio2, (B) bio8, (C) bio9, (D) bio13, (E) bio18, (F) bio19. [file Image5.TIF]

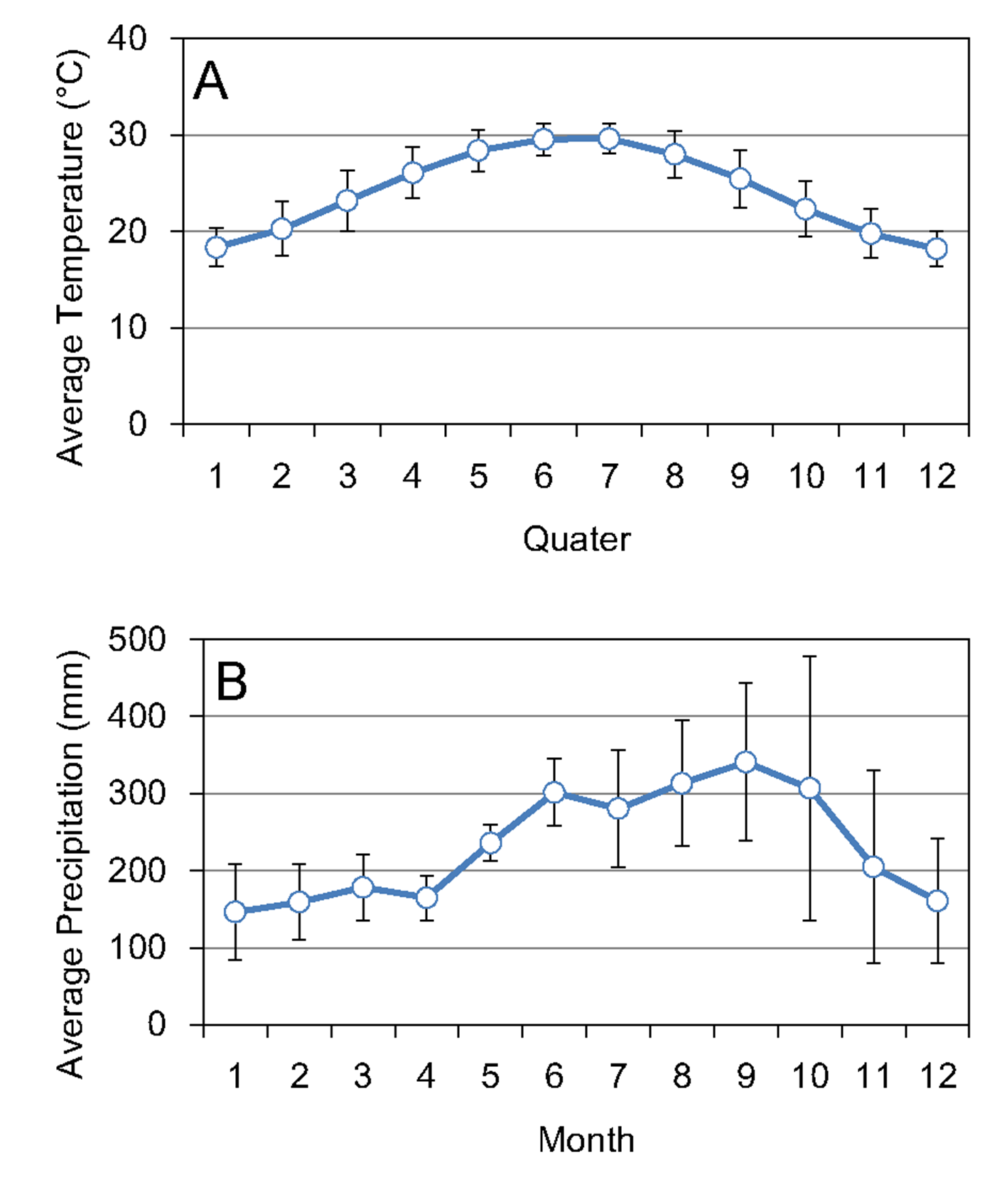

Supplement: Supplementary Figure 6 — The average temperature of every quarter (three months) (A) and the average precipitation of every month (B) of the records of the distribution of bar and tpe. [file Image6.TIF]
